# The Immunoproteasome Subunits LMP2, LMP7 and MECL-1 Are Crucial Along the Induction of Cerebral Toxoplasmosis

**DOI:** 10.3389/fimmu.2021.619465

**Published:** 2021-04-21

**Authors:** Timothy French, Nicole Israel, Henning Peter Düsedau, Anne Tersteegen, Johannes Steffen, Clemens Cammann, Eylin Topfstedt, Daniela Dieterich, Thomas Schüler, Ulrike Seifert, Ildiko Rita Dunay

**Affiliations:** ^1^ Institute of Inflammation and Neurodegeneration, Otto-von-Guericke-University Magdeburg, Magdeburg, Germany; ^2^ Friedrich Loeffler-Institute of Medical Microbiology-Virology, University Medicine Greifswald, Greifswald, Germany; ^3^ Institute of Molecular and Clinical Immunology, Otto-von-Guericke-University Magdeburg, Magdeburg, Germany; ^4^ Institute of Pharmacology, Otto-von-Guericke-University Magdeburg, Magdeburg, Germany; ^5^ Center for Behavioral Brain Sciences, Magdeburg, Germany

**Keywords:** Toxoplasma, immunoproteasome, neuroinflammation, cerebral toxoplasmosis, LMP

## Abstract

Cell survival and function critically relies on the fine-tuned balance of protein synthesis and degradation. In the steady state, the standard proteasome is sufficient to maintain this proteostasis. However, upon inflammation, the sharp increase in protein production requires additional mechanisms to limit protein-associated cellular stress. Under inflammatory conditions and the release of interferons, the immunoproteasome (IP) is induced to support protein processing and recycling. In antigen-presenting cells constitutively expressing IPs, inflammation-related mechanisms contribute to the formation of MHC class I/II-peptide complexes, which are required for the induction of T cell responses. The control of *Toxoplasma gondii* infection relies on Interferon-γ (IFNγ)-related T cell responses. Whether and how the IP affects the course of anti-parasitic T cell responses along the infection as well as inflammation of the central nervous system is still unknown. To answer this question we used triple knockout (TKO) mice lacking the 3 catalytic subunits of the immunoproteasome (β1i/LMP2, β2i/MECL-1 and β5i/LMP7). Here we show that the numbers of dendritic cells, monocytes and CD8^+^ T cells were reduced in *Toxoplasma gondii*-infected TKO mice. Furthermore, impaired IFNγ, TNF and iNOS production was accompanied by dysregulated chemokine expression and altered immune cell recruitment to the brain. T cell differentiation was altered, apoptosis rates of microglia and monocytes were elevated and STAT3 downstream signaling was diminished. Consequently, anti-parasitic immune responses were impaired in TKO mice leading to elevated *T. gondii* burden and prolonged neuroinflammation. In summary we provide evidence for a critical role of the IP subunits β1i/LMP2, β2i/MECL-1 and β5i/LMP7 for the control of cerebral *Toxoplasma gondii* infection and subsequent neuroinflammation.

## Introduction

*Toxoplasma gondii* (*T. gondii*) is a highly successful intracellular parasite capable of infecting all mammals including around 30-70% of all humans (1). In humans, *T. gondii* infection is usually asymptomatic and resolves with minimal pathology. However, if infected individuals acquire an immunodeficiency with impaired T cell function later in life, they are at risk for reactivation of latent toxoplasmosis (2). Early control of *T. gondii* is dominated by innate immune cells such as macrophages, dendritic cells (DCs) and circulating monocytes as well as their secreted proinflammatory cytokines, e.g. tumor necrosis factor (TNF) and interleukin (IL)-12 (3–5). Interferon-γ (IFNγ) is essential for the cell-mediated control of *T. gondii*. Its production by natural killer (NK) cells and T cells is induced by TNF and IL-12 (6). Moreoever, two major mechanisms involved in parasite control are the IFNγ-induced activation of myeloid cells and cytotoxic activity of CD8^+^ T cells (7). IFNγ induces inducible nitric oxide synthase (iNOS) expression by myeloid cells which in turn promotes the production of nitric oxide (NO) thereby inhibiting parasite growth (8). CD8^+^ T cells are known to be crucial for long-term control and containment of *T. gondii*. They prevent the transformation of cyst-forming bradyzoites into fast-replicating tachyzoites thereby achieving both, a restriction of parasite burden as well as the establishment of chronic infection (9, 10). CD8^+^ T cell-derived IFNγ is crucial for long term disease control and relies on CD4^+^ T cell help to facilitate antigen-presentation and upregulate co-stimulatory molecule expression on antigen-presenting cells (APCs). In order to maintain a stable anti-parasite CD8^+^ T cell response, APCs must present parasite-derived peptides *via* major histocompatibility complex class I (MHC I) (11, 12). This requires intracellular processing of parasite proteins, a mechanism which is mainly mediated by the immunoproteasome (IP), a proteolytic protein complex which is induced upon inflammation, e.g. by IFNγ (7, 13).

Upon IFNγ stimulation, standard proteasomes are replaced by *de-novo* synthesized IPs, harboring the three catalytically active subunits β1i/LMP2, β2i/MECL-1 and β5i/LMP7 instead of β1/delta, β2/zeta and β5/MB1. In cells of hematopoietic origin IPs are constitutively expressed (14). In APCs IP expression results in the generation of an altered peptide repertoire and increased number of MHC I ligands due to enhanced protein substrate turnover and changed cleavage specificities (15–17). Whether and how the simultaneous absence of the inducible catalytic subunits β1i/LMP2 (*Psmb8*), β2i/MECL-1 (*Psmb9*) and β5i/LMP7 (*Psmb10*) alters the course of infections remains unclarified.

Research exploring IP function in inflammatory diseases of the central nervous system (CNS) has largely focused on stroke and Alzheimer’s disease (18, 19), where a marked upregulation of IP in reactive glia has been described. The IP is also associated with an increase in phagocytosis and iNOS production in microglia, a common feature of many neurodegenerative diseases (20–22). To better understand how the IP functions in the CNS and especially during neuroinflammation, infection models are sorely needed. Upon LCMV infection in the CNS, LMP7 was vital for the CD8^+^ T cell-induced pathogenesis of LCMV-induced meningitis as LMP7^-/-^ mice exhibited a reduced and delayed disease outcome with fewer infiltrating immune cells (23). Interestingly, this seemed to be LMP7 specific, as LMP2^-/-^ and MECL-1^-/-^ mice had no change in disease compared to WT mice.

In regards to the IP’s role during *T. gondii* infection, previous work from Tu et al., described that mice absent of the single subunits LMP2 or LMP7 were more susceptible to acute *T. gondii* infection (24). Primarily investigating the effect of the IP on the induction of a Th1 immune response, they observed that the acute stage of the infection with fast replicating tachyzoites strongly upregulated the expression of both IP subunits, LMP2 and LMP7, in APCs collected from peritoneal exudate cells (PEC). Further, LMP7^-/-^ mice exhibited strong DC dysfunction as their ability to present immunogenic peptides was impaired and the subseqeunt CD8^+^ T cell IFNγ and Granzyme B response was significanlty reduced compared to WT counterparts. Of note, there was little observable change in these cell types in LMP2^-/-^ mice in the periphery, however, these mice were still susceptible to *T. gondii* infection.

In order to investigate the role of the IP through the course of CNS infection-induced inflammation, we assessed how the absence of all three catalytic IP subunits in TKO mice affects the course of infection-induced inflammation using the neurotropic parasite *T. gondii*. Hereby, we investigated IP deficiency over the course of *T. gondii* infection, focusing on its role in the chronic phase of infection, where the encysted parasite resides primarily in the CNS. This study shows for the first time a prolonged neuroinflammation that is maintained by perturbed cytokine release due to chronic *T. gondii* infection. In addition, we demonstrate increased production of iNOS in microglia and myeloid subsets in brain tissue of infected TKO animals as well as reduced numbers of regulatory T cells, reduced STAT3 phosphorylation but increased induction of apoptosis in myeloid cells. This study demonstrates that IP deficiency results in a lack of parasite control by ultimately increasing susceptibility of these animals to *T. gondii*, highlighting the importance of the IP in terms of induction, maintenance and resolution of *T. gondii*-induced neuroinflammation.

## Methods

### Animals

Conventional immunoproteasome Triple KO (TKO) mice C57BL/6J-LMP2/*Psmb9* ^-/-^MECL-1/*Psmb10* ^-/-^LMP7/*Psmb8* ^-/-^ were kindly provided by Prof. Kenneth L. Rock and Regeneron Pharmaceuticals, Inc. (VG MAID number VG1230 + *Psmb10*) (15). 8 to 12 week-old C57BL/6J mice were bred in the same animal facility. Mice were age and sex matched between the wild type (WT) and deficient mice. All mice were group-housed in 12-h day/night cycles at 22 °C with free access to food and water. All animal experiments were approved by local authorities according to German and European legislation.

### *Toxoplasma gondii* Infection

*T. gondii* cysts of type II strain ME49 were harvested from brains of female NMRI mice chronically infected with *T. gondii* cysts 6-10 months earlier, as described previously (25). In short, isolated brains were mechanically homogenized in 1 ml sterile phosphate-buffered saline (PBS), and the number of cysts in the homogenate was determined using a light microscope. Mice were infected with two cysts *via* oral gavage.

### Organ Collection

First, mice were deeply anaesthetized by isoflurane inhalation (Baxter). Subsequently, mice were transcardially perfused with 60 ml sterile PBS. Single-cell suspension of mesenteric lymph nodes and spleen were generated by mechanically passing tissue through a 40 μm strainer in PBS complemented with 2% fetal calf serum (FCS). Brains were removed and stored in RPMI medium (life technologies) or RNA*later* (Qiagen) for additional analysis. Samples stored in RNA*later* were kept at 4 °C overnight and then transferred to -20°C. Samples in RPMI medium were stored on ice until further experimental procedures.

### Cell Isolation

To isolate brain immune cells, brains were homogenized in a buffer containing 1 M HEPES (pH 7.3) and 45 % glucose and then filtered through a 70 µm strainer. Leukocytes were separated *via* Percoll density gradient centrifugation (GE Healthcare) as we described previously (26). Living cells were counted using a Neubauer counting chamber and trypan blue staining.

### Flow Cytometric Analysis

Single cell suspensions were incubated with an anti-FcγIII/II receptor antibody (clone 93, eBioscience) to block unspecific binding and Zombie NIR™ (BioLegend), a fixable viability dye. Thereafter, cells were stained with fluorochrome-conjugated antibodies against cell surface markers: CD45 (30-F11), CD11b (M1/70), Ly6C (HK1.4), CD45.2 (104), CD40 (3/23), MHCI (28-14-8) and MHCII (M5/114.15.2) all purchased from eBioscience; CD3 (17A2), CD4 (RM4-5), CD8α (53-6.7), CD80 (16-10A1), CD44 (IM7), CD62-L (MEL-14), PD-1 (29F.1A12) and NK1.1 (PK136) all purchased from BioLegend; and Ly6G (1A8) purchased from BD Biosciences in FACS buffer (with 2% FBS, 0.1% NaN3) at 4 °C for 30 min and then fixed in 4% paraformaldehyde (PFA, Affymetrix) for 15 min. Matched FMO controls were used to assess the level of background fluorescence in the respective detection channel.

Intracellular staining was performed on 5x10^5^ cells/well after *ex vivo* stimulation with *Toxoplasma* lysate antigen (200 µg/mL) in the presence of brefeldin A (10 µg/mL, BioLegend) and monensin (10 µg/mL, BioLegend) at 37 °C for 6 h. Afterwards, cells were incubated with anti-FcγIII/II receptor antibody (clone 93, eBioscience) and Zombie NIR™ (BioLegend). Surface epitopes were then stained with CD45 (30-F11), CD11b (M1/70), Ly6C (HK1.4), Ly6G (1A8), CD3 (17A2), CD4 (RM4-5) and CD8α (53-6.7) for 30 min at 4 °C. Stained cells were fixed in 4% PFA and permeabilized using Perm/Wash Buffer (BioLegend). To measure cytokine expression, cells were stained with the flourochrome-conjugated antibodies against intracellular proteins TNF (MP6-XT22), FoxP3 (FJK.16s) and IL-12p40 (C17.8) purchased from eBioscience; iNOS (clone 6, BD Biosciences), Granzyme B (QA16AO2, BioLegend), and IFNγ (XMG1.2, BioLegend) in permeabilization buffer (Invitrogen) for 45 min. Matched isotype controls were used to assess the level of non-specific binding. Flow cytometric analysis was performed on BD LSRFortessa (BD Bioscience) and on Attune NxT Flow Cytometer (Thermo Fisher) and analyzed with FlowJo (version 10, Flowjo LLC).

Calculation of absolute cell count was performed by multiplying the viable population frequencies derived from flow cytometry analysis with the hemocytometer cell count of the respective sample.

### Apoptosis Assay

Cellular apoptosis was quantified using a FITC Annexin V Apoptosis Detection Kit with 7-AAD (BioLegend) following the manufacturer’s instructions. 5x10^5^ splenocytes were isolated, as described above, rinsed with staining buffer and resuspended in Annexin V Binding Buffer (BioLegend). The cells were then incubated with 5 µL of FITC Annexin V and 10 µL of 7-AAD solution for 20 min at room temperature light protected. Fluorescence was measured on Attune NxT Flow Cytometer (Thermo Fisher) and analyzed with FlowJo (version 10, Flowjo LLC).

### Transwell CD8^+^ T Cell Migration Assay

Naïve CD8^+^ T cells were purified using CD8α T Cell Isolation Kit mouse (Miltenyi Biotec) following the manufacturer’s instruction. Chemokines CXCL12 and CCL21 (Peprotech) were used at 250 ng/mL each in 500 μL of Assay Medium containing RPMI 1640, 10mM HEPES and 0.1% BSA (Applichem). Migration assay was performed by seeding 2x10^6^ cells in 200 µL Assay Medium into the upper chamber of 48-well transwell plates (Corning) with a pore size of 5 µm. Strainer was pre-coated with poly-L-lysine (1:100 in PBS) for 20 min at 37 °C prior to the experiment. Following 2.5 h of incubation at 37 °C and 5% CO_2_, cells were collected from the lower chamber and analyzed using MACSQuant^®^ Analyzer (Miltenyi Biotec). Total migrated cells of control mice were set to 100% and relative migration of CD8^+^ T cells from TKO mice was calculated.

### Western Blot

Proteins of whole brain lysates were analyzed by immunoblotting against β1i/LMP2 gp, β5i/LMP7 rb (both custom-generated), β2i/MECL-1 [K65 rb; (27)] and β-Actin (#A1978, Sigma-Aldrich).

Tibias and femurs of 10-14 weeks-old WT and TKO mice were aseptically removed, and bone marrow cells were flushed out with sterile PBS and centrifuged at 150 ×g for 10 min. Cells were resuspended in RPMI medium containing 10% FCS (Capricorn), recombinant murine granulocyte-macrophage colony-stimulating factor (2 ng/ml; Cell Signaling Technology) and 50 μM mercaptoethanol (Sigma-Aldrich) and cultivated for at least 10 days at 37 °C and 5% CO_2_. Twenty-four hours prior to experiments, cells were harvested by scraping and seeded into 6-well plates. For investigation of signaling events cells were treated for the depicted time points with 30 µg/ml *Toxoplasma* lysate Antigen (TLA) and harvested using Trizol reagent (Invitrogen). Proteins were quantified *via* Bradford assay and subsequently analyzed by immunoblotting against pStat3 (Tyr705) (D3A7; XP^®^ Rabbit mAb #9145 CST), Stat3, pMEK (Ser217/221) (41G9; Rabbit mAb #9154 CST), pErk (Thr202/Tyr204) (20G11; Rabbit mAb #4376 CST), Erk and GAPDH (all Cell Signaling Technology) antibodies.

### DNA and RNA Isolation

Samples stored in RNA*later* were homogenized in BashingBeads tubes (Zymo Research, Freiburg, Germany). AllPrep DNA/RNA Mini Kit (Qiagen) was used to isolate DNA and the peqGOLD total RNA kit (Peqlab, Erlangen, Germany) was used to isolate total RNA from the homogenate following the manufacturer’s instructions.

### Semiquantitative RT-qPCR

*T. gondii* burden was determined using the FastStart Essential DNA Green Master kit (Roche). The target *T. gondii* gene used was *Tgb1*, and *Mm. Asl* (TIBMolbiol, Berlin, Germany) was used as a reference gene. The stage of parasite burden was quantified using the Power SYBR^®^ Green RNA-to-CT™ 1-Step Kit (Thermo Fisher) for bradyzoite-specific Bag1 and tachyzoite-specific Sag1 using *Gapdh* as reference gene. All genes were purchased from TIBMolbiol, Berlin, Germany.

Relative gene expression was determined similar to previous descriptions (28, 29) using the TaqMan^®^ RNA-to-CT™ 1-Step Kit (life technologies). TaqMan^®^ Gene Expression Assays (life technologies) were used for mRNA amplification of *Psmb8* (Mm00440207_m1), *Psmb9* (Mm00479004_m1), *Psmb10* (Mm00479052_g1), *Ccl2* (Mm00441242_m1), *Ccl3* (Mm00441259_g1), *Cxcl2* (Mm00436450_m1), *Cxcl10* (Mm00445235_m1), *Ifng* (Mm00801778_m1), *Tnf* (Mm00443258_m1), *Il12a* (Mm00434165_m1), *Nos2* (Mm00440485_m1). Expression of *Hprt* (Mm01545399_m1) was chosen as reference and target/reference ratios were calculated with the LightCycler^®^ 96 software version 1.1 (Roche). All results were further normalized to the mean of the WT infected group.

### Cytokine and Chemokine Assessment

Cytokine and chemokine profile was characterized using the LEGENDplex™ system (BioLegend). A more detailed protocol is published (30). Briefly, we used the Mouse Inflammation Panel (13-plex) system. Serum from WT and TKO mice was collected and incubated with fluorescence-encoded capture beads to cytokine and chemokine targets including CCL2, TNF and IFNγ. The fluorescent signals of analyte-specific bead regions were quantified using flow cytometry, and the concentrations of particular analytes were determined using provided data analysis software (BioLegend, LegendPlex™ software v8.0).

### Statistical Analysis

Datasets were analyzed statistically using GraphPad Prism 7.02 (Graphpad software). To test for significance, we used a Mann-Whitney test for comparing two groups and a 2way ANOVA with uncorrected Fischer’s LSD test for multiple comparisons. Owing to the small sample sizes, unequal variances were assumed in all *t*-tests. The significance level was set to P < 0.05 for all statistical comparisons. Symbols represent individual animals, columns represent mean values and error bars represent **±** SEM.

## Results

### TKO Mice Show Increased Susceptibility to *T. gondii* Infection

**Figure 1:**
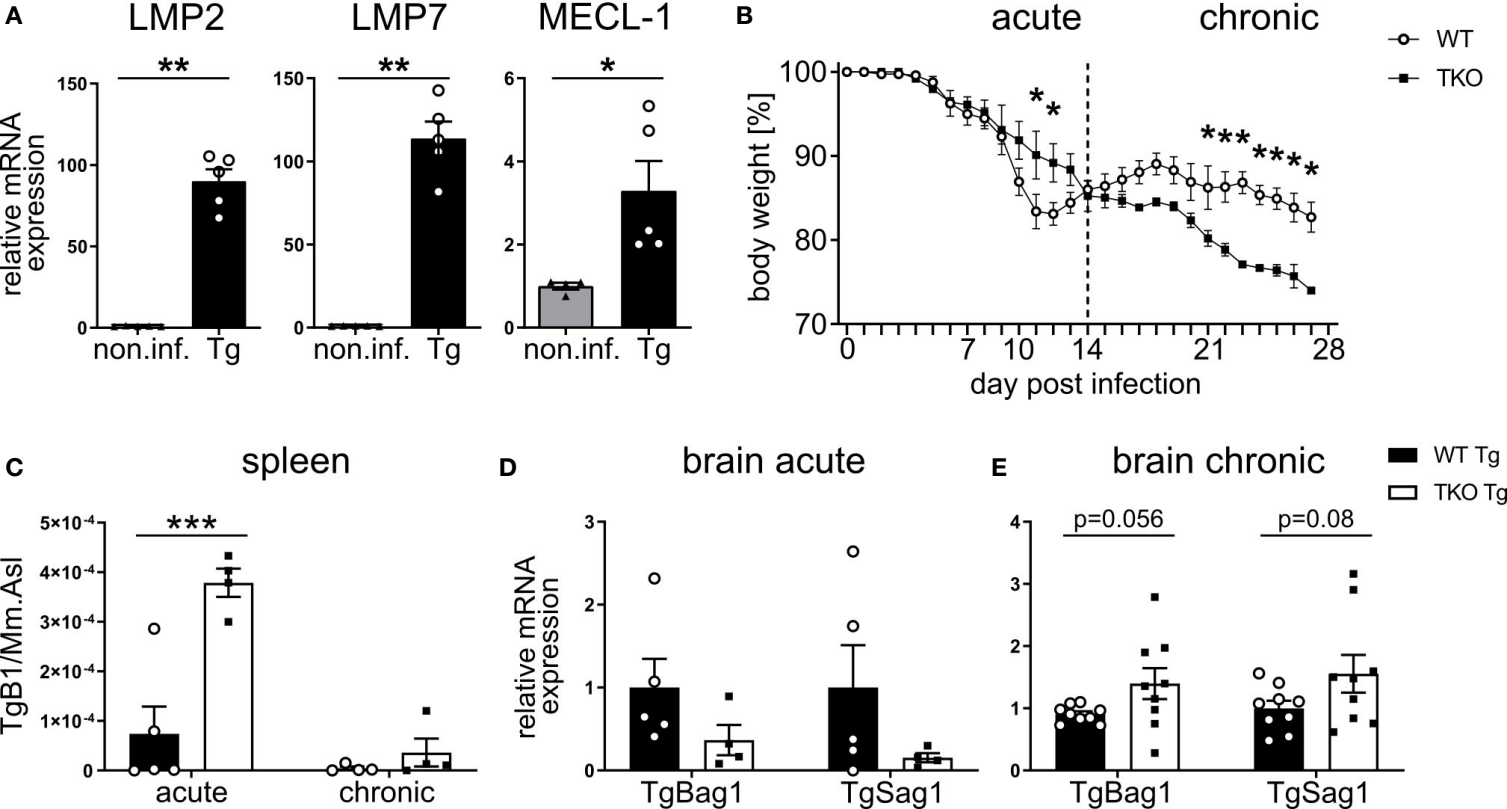
Increased susceptibility of TKO mice in the chronic, but not acute, phase of *T. gondii* infection. Wild type (WT) mice were orally infected with a low dose (2 cysts) of *T. gondii* (ME49) for 28 days. Brains were collected from WT non-infected (non.inf., n ≥ 4) and *T. gondii* infected (Tg, n ≥ 4) animals on day 28 *p.i.* and following homogenization, mRNA was extracted for RT-qPCR analysis. **(A)** mRNA expression of the immunoproteasome subunits (LMP7/*Psmb8*, LMP2/*Psmb9*, MECL-1/*Psmb10*) were normalized to the non-infected group. Data is representative of four independent experiments. **(B)** WT mice and triple-knocked out (TKO) for the immunoproteasome subunits (LMP7/Psmb8^-/-^LMP2/Psmb9^-/-^MECL-1/Psmb10^-/-^) mice were orally infected with a low dose (2 cysts) of *T. gondii* and weighed daily. Day 10 and 28 *p.i.* were chosen as time points for the acute and chronic immune response. The spleens and brains were taken from acute (d10 *p.i.*) and chronic (d28 *p.i.*) *T. gondii*-infected WT (WT Tg, n=4) and triple-knockout (TKO Tg, n=4) mice. Organs were homogenized and DNA/RNA was isolated from each for qPCR and RT-qPCR analysis. **(C)** qPCR analysis from DNA extracted from spleens of *T. gondii* infected WT and TKO mice. Relative quantification of *T. gondii* gene *TgB1* in spleen from acute (d10 *p.i.*) and chronic (d28 *p.i.*) *T. gondii* infected WT and TKO mice. *TgB1* gene expression was normalized to the gene expression of the reference gene *Mm.Asl*. **(D, E)** RT-qPCR analysis from RNA extracted from brain homogenates of mice from the acute (d10 *p.i.*) and chronic (d28 *p.i.*) phase of infection. Relative mRNA levels were normalized to the mean expression of the infected WT group. Data shown in **(A)** represents three independent experiments and data shown in **(B–E)** represent four independent experiments. In **(A, C–E)** symbols represent individual animals, columns represent mean values and error bars represent ± SEM. In **(B)**, data points represent mean values and error bars represent ± SEM. In **(A)**, a Mann-Whitney test for two groups and in **(B–E)** a 2way ANOVA following Fisher’s LSD test was used for statistical analysis. *P < 0.05, **P < 0.01, ***P < 0.001.

The 20S catalytic core particle of the IP consists of multiple subunits, three subunits harbor the six active sites that differ from those in the standard proteasomes. The relative contribution of immunoproteasomes to immune responses against *T. gondii* is unclear. To determine the relative expression of the three IP catalytic-subunits LMP2 (*Psmb9*), LMP7 (*Psmb8*) and MECL-1 (*Psmb10*) during the acute and chronic neuroinflammatory stage of infection, mRNA and protein was isolated from brain homogenates of *T. gondii* infected wild type (WT) mice at day 28 post-infection (*p.i.*). As compared to uninfected controls, the expression of all three IP subunits LMP2 (*Psmb9*), LMP7 (*Psmb8*) and MECL-1 (*Psmb10*) was significantly increased in *T. gondii* infected WT mice both on the RNA and protein level (**Figure 1A** and **Supplementary Figure 1**). To investigate the functional significance of these IP subunits we used mice with a combined deficiency of LMP2, MECL-1 and LMP7. These triple-knockout (TKO) mice and WT controls were infected with *T. gondii* orally (*p.o.*) and body weight was monitored daily throughout the course of the infection (**Figure 1B**). During the acute phase of infection, from day 10 to 14 *p.i.*, WT mice showed a higher weight loss when compared to TKO mice. Starting around day 13 *p.i.*, however, this effect was reversed and bodyweight loss was significantly more pronounced in TKO mice from day 21 to 28 *p.i.* Parasite burden was significantly increased in the spleen of TKO mice already at day 10 *p.i.*, an effect that was not observed at day 28 *p.i.* (**Figure 1C**). This might be due to the fact that *T. gondii* invades deeper tissues including the brain to evade the hosts’ immune system (31).

Consequently, we analyzed parasite burden in the brain. To assess differences in stage conversion of the fast replicating tachyzoite and slow replicating bradyzoite stages of *T. gondii*, we utilized *T. gondii*-specific genes (TgSAG1 and TgBAG1, respectively). We detected a reduced mRNA expression of both tachyzoites and bradyzoites genes in brains of infected TKO mice in the acute phase of infection (**Figure 1D**), but increased mRNA expression in the chronic phase of infection (**Figure 1E**). Hence, altered tissue distribution of *T. gondii* in TKO mice argues for impaired peripheral immune responses in the absence of a functional IP.

#### Reduced/Delayed Type 1 Immune Response to *T. gondii* in TKO Mice

**Figure 2:**
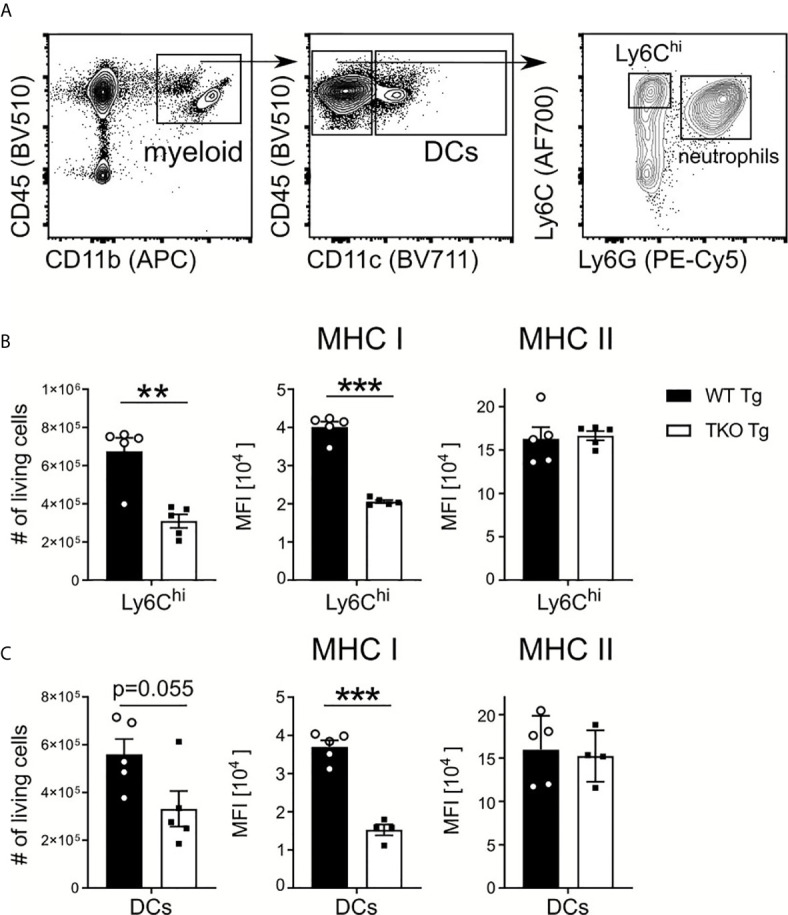
Reduced numbers of Ly6C^hi^ monocytes and DCs in spleen of infected TKO mice. Immune cells were isolated from the spleens of *T. gondii* infected WT (WT Tg, n=5) and TKO (TKO Tg, n ≥ 4) mice on day 10 *p.i.* and analyzed by flow cytometry. Following viability staining and the basic FSC/SSC gating, viable single cells were chosen for further characterization. **(A)** Splenocytes were first gated based on surface expression of CD45, a hematopoietic marker, and CD11b, a myeloid cell marker (left plot). CD11b^+^CD45^+^ cells were further gated for CD11c and CD11c^+^ cells identified as dendritic cells (DCs) (center plot). CD11c^-^ cells were further divided into inflammatory monocytes (Ly6G^-^Ly6C^hi^) and neutrophils (Ly6G^+^) (right plot). The total number of living cells and surface expression of MHC I and MHC II were assessed for Ly6C^hi^ monocytes **(B)** and DCs **(C)**. Expression of MHC I and MHC II was quantified using the mean fluorescence intensity (MFI) of their respective fluorochrome. Data shown in **(A)** is a representative of three independent experiments. Data shown in **(B**, **C)** represent three independent experiments; symbols represent individual animals, columns represent mean values and error bars represent ± SEM. A Mann-Whitney test was used for statistical analysis. **P < 0.01, ***P < 0.001.

Early immune responses against *T. gondii* strongly depend on the pathogen-associated molecular pattern (PAMP)-dependent activation of APCs. They produce TNF and IL-12, promote the activation of NK and T cells, which produce anti-parasitic IFNγ (6). To determine if the IP affects early parasite recognition in the periphery, splenic Ly6C^hi^ inflammatory monocytes and DCs from WT and TKO mice were analyzed in the acute phase of infection. As shown in **Figures 2A–C**, numbers and MHC I levels of Ly6C^hi^ inflammatory monocytes and DCs were significantly reduced in the spleen of infected TKO compared to WT mice. In contrast, MHC II expression proved to be independent of the IP which is consistent with previously published data (15).

**Figure 3:**
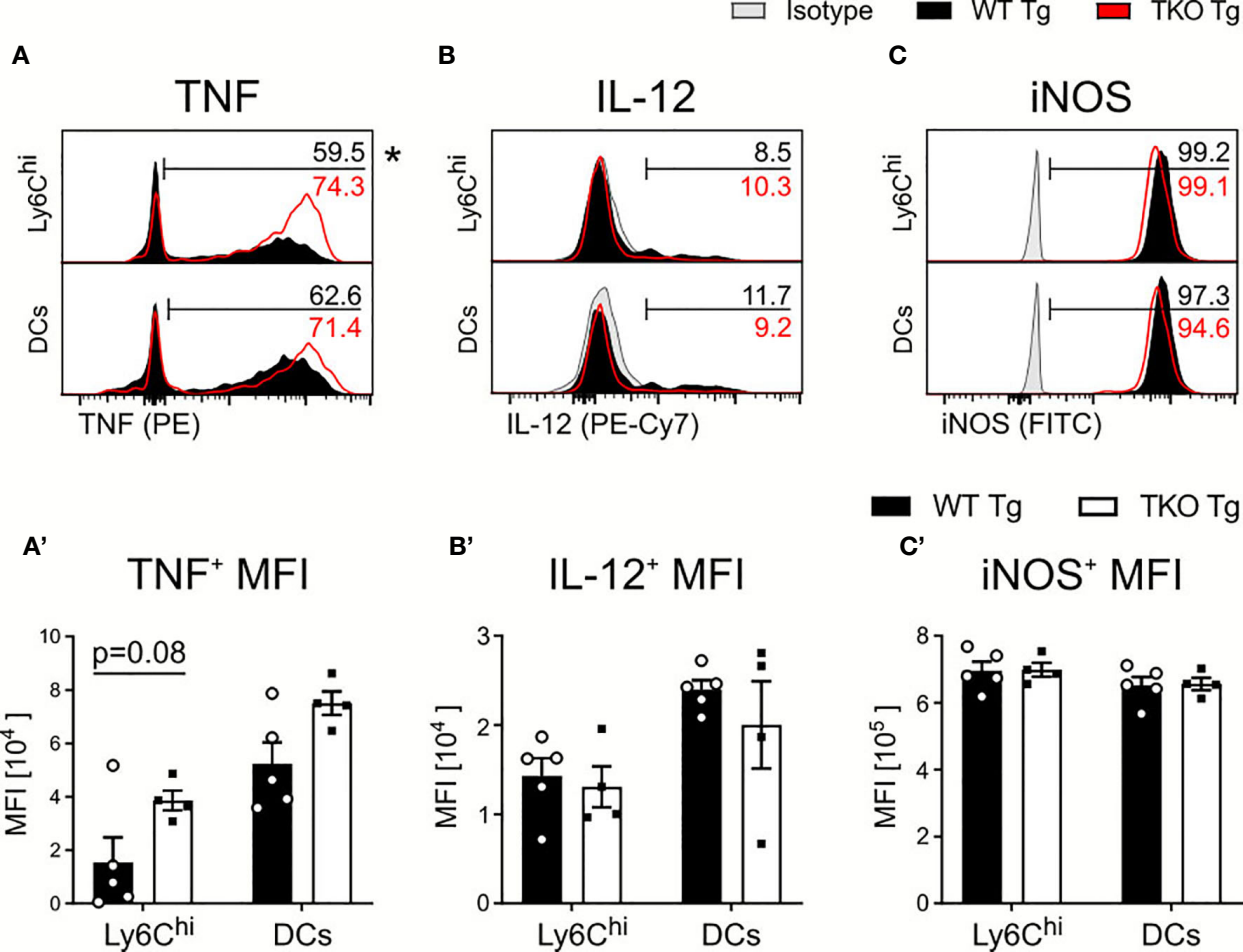
Cytokine production by APCs in spleens of *T. gondii* infected mice. Immune cells were isolated from the spleens of *T. gondii* infected WT (WT Tg, n=5) and TKO (TKO Tg, n=4) mice on day 10 *p.i.* Isolated cells were then restimulated with *T. gondii*-lysate antigen (TLA) for 6 hours, stained and analyzed by flow cytometry. **(A–C)** Histograms of Ly6C^hi^ monocytes and DCs intracellular production of **(A)** TNF **(B)** IL-12 and **(C)** iNOS and their resulting MFI expression **(A’–C’)**. The histogram values (right side) represent the average percentage of positively expressing cells (determined by isotype control; in gray) for each respective immune marker and group (WT in black; TKO in red outline). The bar **(A–C)** outlines where positive expression begins for each respective cell and marker. Data shown in **(A–C)** are representatives of three individual experiments. Data shown in **(A’–C’)** represent three independent experiments; symbols represent individual animals, columns represent mean values and error bars represent ± SEM. 2way ANOVA following Fisher’s LSD test was used for statistical analysis. *P < 0.05.

Next, we investigated whether IP deficiency affects IL-12 and TNF production by Ly6C^hi^ monocytes and DCs. Upon *ex vivo* restimulation with Toxoplasma lysate antigen (TLA), we observed a significantly higher percentage of Ly6C^hi^ monocytes producing TNF with increased TNF production and non-significant change in frequencies of TNF producing DCs (**Figures 3A**, **A’**) in TKO mice in the acute phase. We detected no difference in the percentage of IL-12-producing DCs and Ly6C^hi^ monocytes or in the IL-12 produced (**Figures 3B**, **B’**). TNF and IL-12 production lead to the expression of IFNγ, a key molecule for *T. gondii* elimination (4, 6). IFNγ induces cell-autonomous immune responses (32), such as induction of inducible nitric oxide synthase (iNOS) which produces nitric oxide (NO) thereby promoting parasite clearance (33, 34). As shown in **Figures 3C**, **C’**, iNOS production by DCs and Ly6C^hi^ monocytes was also indistinguishable between infected WT and TKO animals. These results indicate that the IP has only a minor impact on early innate immune responses against the parasite but may be required for IFNγ-related adaptive immune responses.

**Figure 4:**
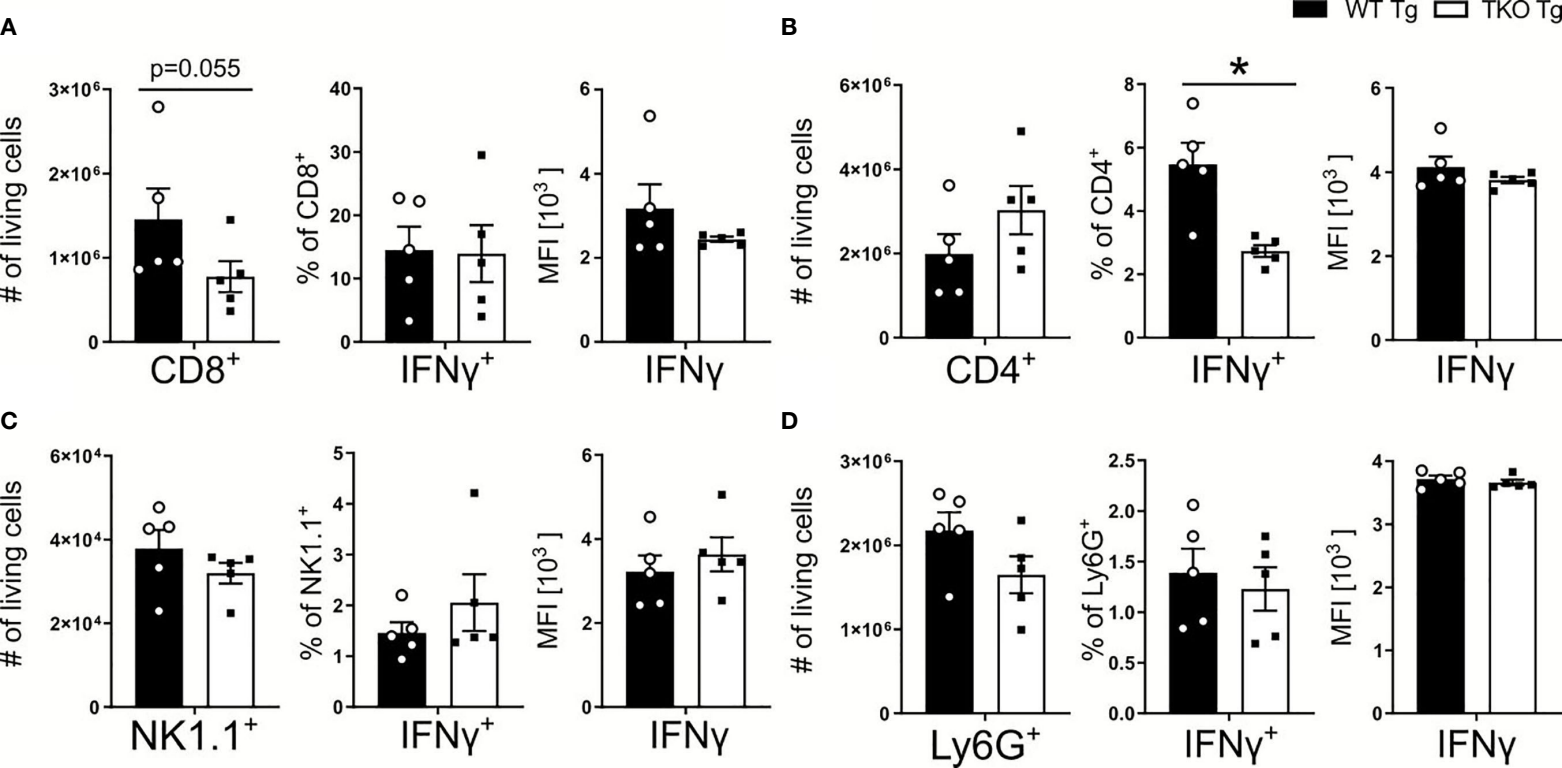
Reduced CD8^+^ T cell numbers and impaired Th1 responses in *T. gondii* infected TKO mice. Immune cells were isolated from the spleens of *T. gondii* infected WT (WT Tg, n=5) and TKO (TKO Tg, n=5) mice on day 10 *p.i.* and analyzed by flow cytometry. Following viability staining and the basic FSC/SSC gating, viable single cells were determined by first removing CD11b^+^ and CD3^-^ immune cells. CD3^+^CD4^+^ and CD3^+^CD8^+^ T cells were identified for further analysis. CD45^+^NK1.1^+^ cells were determined after gating out CD3^+^, CD8^+^, Ly6C^+^ and Ly6G^+^ cells. The total cell number of **(A)** CD8^+^ T cells, **(B)** CD4^+^ T cells, **(C)** NK1.1^+^ cells and **(D)** neutrophils, the percentage of IFNγ producing cells and their respective IFNγ production were measured. Data shown represents three independent experiments; symbols represent individual animals, columns represent mean values and error bars represent ± SEM. A Mann-Whitney test was used for statistical analysis. *P < 0.05.

IFNγ produced in the course of *T. gondii* infection facilitates IL-12 production by DCs and monocytes (35). With an increased parasite burden in spleens of TKO mice, one would expect increased expression of IL-12. However, we detected no change in IL-12 production (**Figures 3B**, **B’**). In order to characterize IFNγ production by immune cells, CD8^+^ and CD4^+^ T cells, NK1.1^+^ cells and neutrophils were restimulated with TLA *ex vivo* and analyzed by flow cytometry. Fewer CD8^+^ T cells were isolated from the spleens of infected TKO animals compared to WT mice during the acute phase of infection (**Figure 4A**). This, together with the observed reduced MHC I expression on APCs (**Figure 2**) is in line with previously reported results (15, 24, 36) describing reduced CD8^+^ T cell numbers when MHC I/peptide presentation is impaired (24). In addition, we observed slightly elevated numbers of CD4^+^ T cells in spleens of infected TKO mice, a finding that was already visible in naïve TKO mice (**Figure 4B** and **Supplementary Figures 2A–C, E, F**). Consistent with previous findings (37), significantly reduced frequencies in the IFNγ^+^CD4^+^ T cells were detected in spleens of infected TKO mice, whereas steady state analyses revealed no difference in the circulating IFNγ in WT and TKO mice (**Figure 4B** and **Supplementary Figure 2D**). Neither numbers, nor frequencies of IFNγ producing NK1.1^+^ cells or neutrophils (Ly6G^+^) differed significantly between TKO and WT mice (**Figures 4C, D**).

#### Parasite Dissemination Into the Brain of WT and TKO Mice in the Acute Phase of *T. gondii* Infection

**Figure 5:**
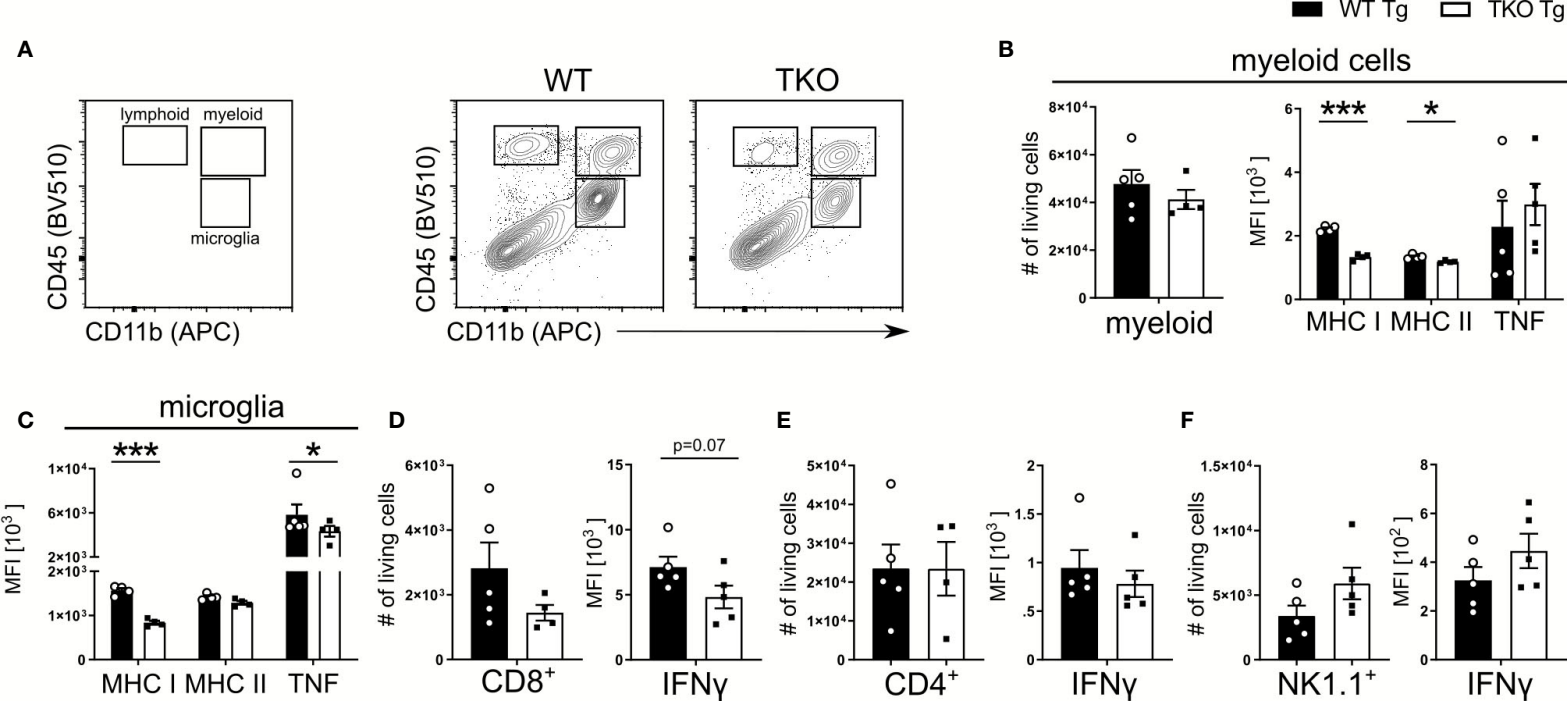
Impaired recruitment and IFNγ-dependent activation of proinflammatory myeloid cells in TKO mice. Immune cells were isolated from brains of *T. gondii* infected WT (WT Tg, n=5) and TKO (TKO Tg, n ≥ 4) mice on day 10 *p.i.* For the measurement of TNF and IFNγ, brain cells were restimulated with TLA for 6 hours and then stained and analyzed by flow cytometry. Following viability staining and the basic FSC/SSC gating, single cells were chosen for further characterization. **(A)** Representative gating strategy using CD45 and CD11b to distinguish between microglia, myeloid cells and lymphoid cells (left plot) and representative plots from from brain tissue of infected WT (center panel) and TKO (right plot) mice. CD11b^+^CD45^int^ cells were identified as microglia and CD11b^+^CD45^hi^ were identified as recruited myeloid cells then divided into Ly6G^-^ monocytes as depicted in Fig 2A. CD45^+^CD11b^-^ cells were divided into CD3^+^CD8^+^ and CD3^+^CD4^+^ T cells. CD45^+^NK1.1^+^ cells were determined after removing CD3^+^, CD8^+^, Ly6C^+^ and Ly6G^+^ cells. Total number of cells was assessed for recruited **(B)** myeloid cells, **(D)** CD8^+^ T cells, **(E)** CD4^+^ T cells and **(F)** NK1.1^+^ cells. Recruited **(B)** myeloid cells and **(C)** microglia were measured for their surface expression of MHC I and MHC II as well as their production of TNF. Recruited **(D)** CD8^+^, **(E)** CD4^+^ and **(F)** NK1.1^+^ cells had their IFNγ production quantified. The expression or production of each immune marker was quantified using the MFI of their respective fluorochromes. Data shown in A is a representative of three independent experiments. Data shown in **(B–F)** represents three independent experiments; symbols represent individual animals, columns represent mean values and error bars represent ± SEM. A Mann-Whitney test for comparing two groups and a 2way ANOVA followed by Fisher’s LSD test for comparing multiple groups was used for statistical analysis. *P < 0.05; ***P < 0.001.

To establish the chronic phase of infection in the CNS, *T. gondii* has to cross the blood-brain barrier (BBB) and enter the brain. When *T. gondii* infects DCs or monocytes, they induce a hypermotility phenotype and enhanced transmigration capacity, effectively shuttling the parasite into the brain, thereby functioning as a Trojan horse to cross the BBB (38). In TKO animals, we observed an increased parasite burden in the periphery but the opposite in the brain on d10 *p.i.* (**Figure 1**). This is also associated with a dysregulated DC and CD8^+^ T cell recruitment to the spleen (**Figures 2** and **4**). To investigate whether and how impaired immune pressure in the periphery corresponds to altered immune cell composition in the brain, we analyzed different immune cell populations in brains of *T. gondii* infected mice on day 10 *p.i.* Using flow cytometry analysis, we assessed recruited myeloid and lymphoid cells into the CNS along with the resident microglia (**Figure 5A**). We observed fewer numbers of myeloid cells recruited into the brain of TKO mice, though not significant (**Figure 5B**). Interestingly, these myeloid cells exhibited a similar phenotype to the peripheral cells (**Figures 2B, C** and **3A**) as they had reduced MHC I expression and normal TNF production (**Figures 5B**, **2B, C**, and **3A**). Nonetheless, myeloid cells in the TKO-brain displayed slightly reduced MHC II expression (**Figure 5B**), which is expected to be due to the reduced presence of parasites in the brain in the acute phase of infection and was not observed on Ly6C^hi^ inflammatory monocytes and DCs obtained from the spleens of TKO mice (**Figures 2B, C**).

*T. gondii* activates resident microglia, which induces the recruitment of immune cells into the brain (39). In infected TKO mice, MHC I and TNF expression by microglia was significantly reduced compared to WT mice (**Figure 5C**). In contrast, circulating TNF was not altered in non-infected TKO mice (**Supplementary Figure 2D**). Furthermore, in the brain of infected TKO mice the size of CD8^+^ T cell pool and availability to produce IFNγ were slightly reduced (**Figure 5D**). The number and IFNγ production of brain CD4^+^ T cells was unchanged whereas the number of NK1.1^+^ cells as well as IFNγ production, were slightly increased in acutely infected TKO mice (**Figures 5E, F**). To directly assess the ability of CD8^+^ T cells to migrate to sites of *T. gondii* infection, we used a transwell migration assay. CD8^+^ T cells were isolated from *T. gondii*-infected WT and TKO mice and stimulated using CCL21 or CXCL12. Interestingly, CD8^+^ T cells from spleens of infected TKO mice showed significantly reduced migration upon both CCL21 and CXCL12 *ex vivo* stimulation compared to WT mice (**Supplementary Figure 3A**). This indicates that CD8^+^ T cells from TKO mice possess a reduced capacity to migrate to the site of infection in the acute phase that suggests a failure of the immune system to limit infection by inducing tachyzoite differentiation into bradyzoites.

An alternative explanation for the reduced pathogen burden in brains of acutely infected TKO mice could be reduced parasite shuttling by myeloid cells, a process which is CCL2-dependent (3, 39). In the serum of infected TKO mice CCL2 levels were slightly, (albeit non-significantly) reduced (**Supplementary Figure 3B**) which aligns with the number of myeloid cells in the brain (**Figure 5B**). Correspondingly, mRNA levels of CCL2 and other myeloid-associated chemokines such as CCL3, CXCL2 and CXCL10 were reduced in brains of infected TKO mice at day 10 *p.i.* (**Supplementary Figure 3C**). This was also the case for IFNγ (**Supplementary Figure 3D**), which is known to induce chemokine gene activity (40). Overall, an absent IP correlates with impaired early induction of adaptive immune responses, leading to a loss of parasite control in the acute phase of infection, subsequently resulting in an increased peripheral parasite burden.

#### WT and TKO Mice During Chronic *T. gondii* Infection

**Figure 6:**
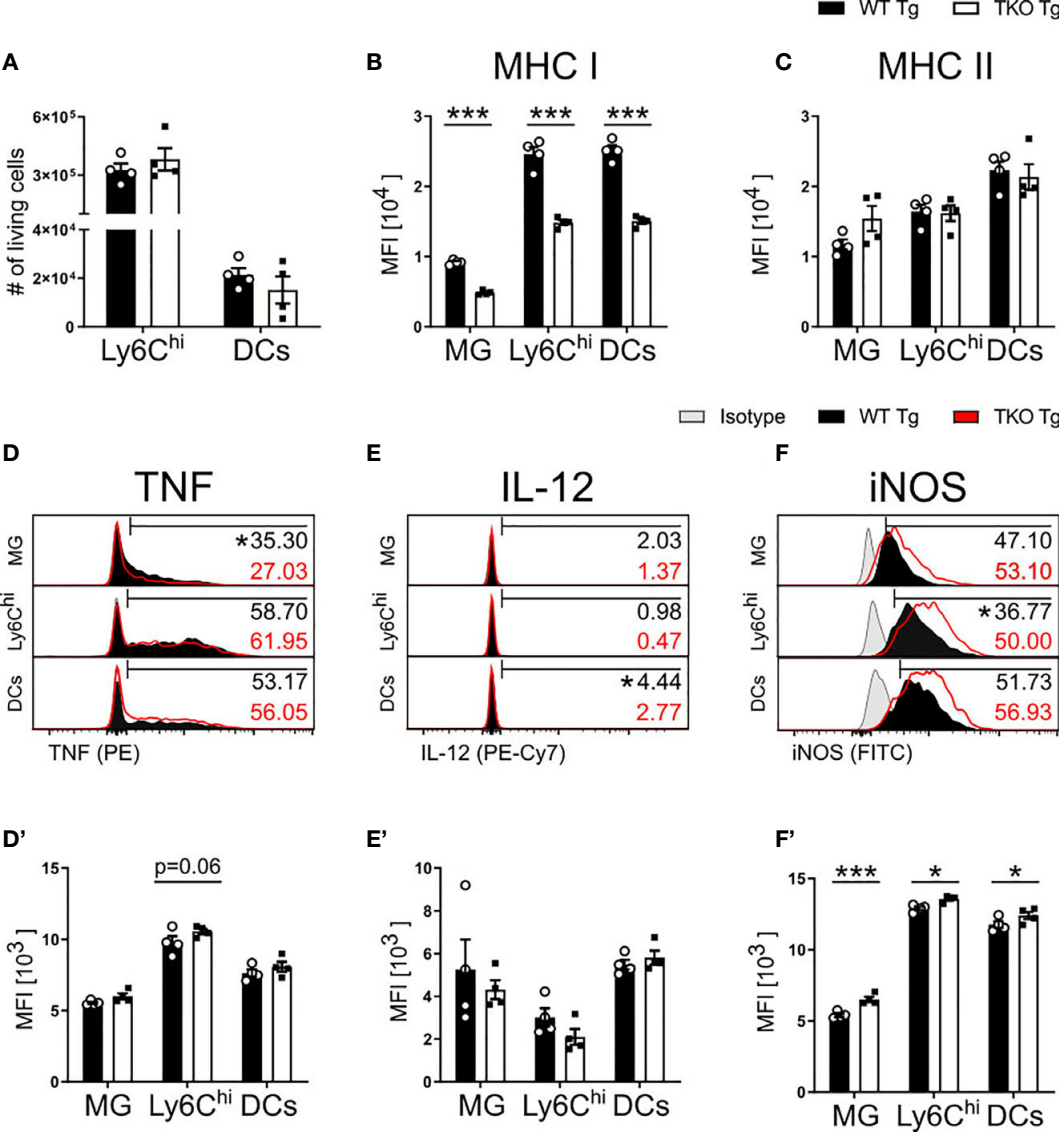
Increased anti-parasitic immune response in brains of TKO mice in chronic stage of infection. Immune cells were isolated from brain homogenate of *T. gondii* infected WT (WT Tg, n=4) and TKO (TKO Tg, n=4) mice on day 28 *p.i.* For the measurement of TNF, IL-12 and iNOS brain cells were restimulated with TLA for 6 hours, stained and analyzed by flow cytometry. Following viability staining and the basic FSC/SSC gating, single cells were chosen for further characterization. Using the same gating strategy as described for Fig 2A and 5A, CD11b^+^CD45^int^ microglia (MG), CD11b^+^CD45^hi^Ly6G^-^Ly6C^hi^ inflammatory monocytes and CD11b^+^CD45^+^CD11c^+^ DCs were analyzed. **(A)** Total cell numbers were calculated as a percentage of live cells found in the brain for Ly6C^hi^ monocytes and DCs. The surface expression of **(B)** MHC I and **(C)** MHC II expression was determined on MG, DCs and Ly6C^hi^ monocytes. Histograms of the intracellular production of **(D)** TNF, **(E)** IL-12 and **(F)** iNOS and their resulting MFI **(D’–F’)**. The histogram values (right side) represent the percentage of positively expressing cells (determined by isotype control; in gray) for each respective immune marker and group (WT in black; TKO in red outline). The bar **(D–F)** outlines where positive expression begins for each respective cell and marker. Data shown represent four independent experiments; symbols represent individual animals, columns represent mean values and error bars represent ± SEM. 2way ANOVA followed by Fisher’s LSD test was performed for statistical analysis. *P < 0.05, ***P < 0.001.

Parasite control during chronic neuroinflammation requires persistent, basal levels of inflammation involving resident microglia and recruited immune cells such as monocytes and T cells. Upon chronic infection, we observed an increased parasite burden in combination with a more severe weight loss in TKO compared to WT mice (**Figure 1E**) that resembled reactivated toxoplasmosis. To further investigate this phenotype, immune cells were isolated from brains of chronically infected mice and analyzed *via* flow cytometry. Ly6C^hi^ inflammatory monocytes and DCs exhibited comparable total numbers in the brains of infected TKO mice (**Figure 6A**). Next, we determined the influence of the IP on the functional capacity of resident microglia and recruited immune cells in chronic inflammation. Again, expression of MHC I continued to be impaired as all cell types exhibited significant reduced expression (**Figure 6B**). Microglia showed a slight increased expression of MHC II in the chronic stage of infection, which is expected with an increased parasite burden (**Figure 6C**). To investigate the effector function of these cells in the chronic stage of infection, we then analyzed their production of TNF, IL-12 and iNOS. Ly6C^hi^ monocytes recruited into the brains of TKO mice showed a trend of increased TNF expression whereas significantly fewer microglia were producing TNF when compared to WT mice (**Figures 6D**, **D’**). Fewer DCs produced IL-12 while no differences in producing microglia or Ly6C^hi^ monocytes could be detected between WT and TKO mice in the chronic stage of infection (**Figure 6E**, **E’**). Interestingly, when assessing iNOS expression in these cell types, they all, especially microglia, showed significantly increased iNOS production in brains of TKO compared to WT mice (**Figures 6F**, **F’**). These results show that in the chronic stage of infection, TKO mice are able to induce IFNγ-driven anti-parasitic immune responses such as the expression of iNOS. Although in TKO mice expression of cell autonomous anti-parasitic effector molecules was induced, they regardless were not able to sufficiently control parasite proliferation in the brain. It is crucial to have *T. gondii* specific T cells that can recognize active, ongoing parasite infection and then prime the local cells to adequately defend and prevent further parasite spread. Thus, we hypothesized that T cells are responsible for the lack of parasite control in the chronic stage of infection and we analyzed T cell responses in chronic inflammation in more detail.

**Figure 7:**
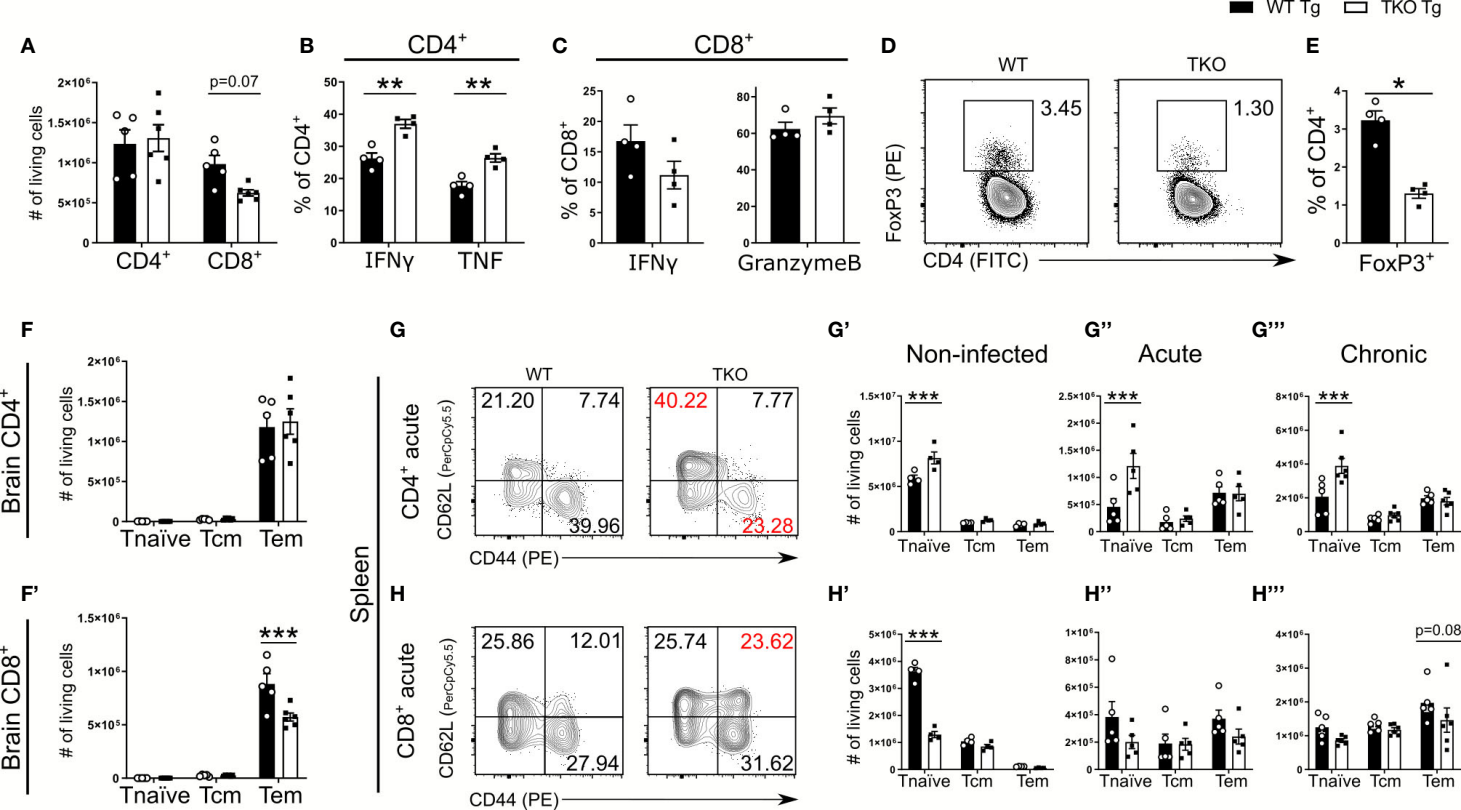
Altered T cell differentiation in infected TKO mice. Immune cells were isolated from brain homogenate of *T. gondii* infected WT (WT Tg, n=4) and TKO (TKO Tg, n=4) mice on day 28 *p.i.* For the measurement of IFNγ, TNF and Granzyme B, cells were restimulated with TLA for 6 hours, stained and analyzed by flow cytometry. **(A)** Total number of CD4^+^ and CD8^+^ T cells recruited to the brain. **(B, C)** Intracellular production of proteins in T cells was characterized by the percentage of cells positive for IFNγ, Granzyme B or TNF. **(D)** Representative gating for regulatory T cells after selecting CD11b^-^CD3^+^ cells. Tregs were determined by gating for CD4^+^FoxP3^+^ cells. **(E)** The frequency of recruited FoxP3^+^ cells was calculated as a percentage of CD4^+^ T cells in the brain. Using CD62L and CD44, CD4^+^ and CD8^+^ T cells were divided into naïve (CD62L^+^CD44^-^), T central memory (T_cm_, CD62L^+^CD44^+^) and T effector memory (T_eff_, CD62L^-^CD44^+^) populations. Total number of differentiated **(F)** CD4^+^ and **(F’)** CD8^+^ T cells from the brains of WT and TKO mice from day 28 *p.i.* **(G, H)** Immune cells were isolated from spleens at steady state, day 10 *p.i.* (acute) and day 28 *p.i.* (chronic) from WT and TKO mice and analyzed by flow cytometry. **(G, H)** Representative gating strategies of these T cell subpopulations for both CD4^+^ and CD8^+^ T cells (acute stage shown). The absolute number of CD4^+^ and CD8^+^ T cells for the respective subpopulations from WT and TKO mice **(G’, H’)** non-infected, **(G’’, H’’)** acute stage infection, **(G’’’, H’’’)** chronic stage infection. Data shown in D, G & H are representatives of three independent experiments. Data shown in **A–C, E, F, F’, G’-G’’’** and **H’–H’’’** represent four independent experiments; symbols represent individual animals, columns represent mean values and error bars represent ± SEM. In C&E a Mann-Whitney test for comparing two groups and in **A, B, F, F’, G–G’’’** and **H–H’’’** a 2way ANOVA followed by Fisher’s LSD test were used for statistical analysis. *P < 0.05; **P < 0.01, ***P < 0.001.

When assessing CD4^+^ and CD8^+^ T cell recruitment into the brain, TKO mice compared to WT mice showed comparable CD4^+^ T cell numbers, but a trend for fewer CD8^+^ T cells (**Figure 7A**). To further assess T cell functionality in response to *T. gondii*, we analyzed IFNγ and TNF production of CD4^+^ T cells as well as IFNγ and Granzyme B secretion by CD8^+^ T cells following *ex vivo* TLA stimulation. Granzyme B is a cytotoxic protein contained in granules of cytotoxic CD8^+^ T cells that is able to induce apoptosis in neighboring infected cells after release. Interestingly, we observed significantly increased frequencies of IFNγ and TNF secreting CD4^+^ T cells in TKO mice compared to WT mice (**Figure 7B**) which is in concordance with our finding that in whole TKO-brains significantly enhanced TNF and non-significantly increased IFNγ mRNA levels can be found (**Supplementary Figure 3E**). Similar to the immune response in the acute phase of infection, TKO mice compared to WT mice showed a lower frequency of IFNγ producing CD8^+^ T cells (**Figure 7C**). Surprisingly, no differences of granzyme B containing CD8^+^ T cells could be detected between TKO and WT mice in brain tissue in the chronic stage of infection (**Figure 7C**). Since it is described that regulatory T cells (Tregs) mediate T cell suppression during the acute phase of *T. gondii* infection, we next analyzed whether TKO mice have changes in the recruitment of Tregs into the CNS. And indeed, we found significantly reduced frequencies of CD4^+^ Tregs in brains of TKO mice compared to WT mice in the chronic phase of infection (**Figures 7D, E**). These results show that the absence of the IP leads to reduced Treg frequencies in the *T. gondii* infection model and subsequent reduced T cell suppression, resulting in increased cytokine production by CD4^+^ T cells (**Figure 7B**).

The immunoproteasome is crucial to induce T cell maturation (41). Thus, we further analyzed different T cells subtypes in respect to their surface expression of CD62L and CD44, allowing us to distinguish between naïve (CD44^-^CD62L^+^), central memory (CD44^+^CD62L^+^) and effector memory (CD44^+^CD62L^-^) T cells. First, we investigated the number of T cell subtypes recruited into the CNS and observed a significant reduction of CD8^+^ T effector memory (T_em_) cells but not CD4^+^ T effector cells in brains of TKO mice in the chronic phase of infection (**Figures 7F**, **F’**). To assess if this significant difference in T cell differentiation is restricted to the chronic infection, we investigated different T cell subtypes of splenocytes in uninfected mice as well as infected mice in the acute and chronic phase of infection (**Figures 7G, H**). Already uninfected TKO mice showed a significant reduction of naïve CD8^+^ T cells and vice versa a significant increase of naïve CD4^+^ T cells in spleen tissue compared to WT mice (**Figures 7G’, H’** and **Supplementary Figures 2B, C**), which is consistent with previous findings (15). We found that TKO mice compared to WT mice had significantly increased numbers of naïve CD4^+^ T cells as well as comparable numbers of central memory T cells (T_cm_) and T_em_ cells throughout the infection (**Figures 7G**, **G’’, G’’’**).

Splenocytes of TKO mice compared to WT mice possessed significantly fewer naïve CD8^+^ T cells in uninfected mice (**Figure 7** **H’**). However, during the course of infection WT and TKO mice had comparable numbers of naïve CD8^+^ T cells (**Figures 7H’’**, **H’’’**), but TKO mice exhibited reduced T_em_ cells in the chronic stage of infection (**Figure 7H’’’**). These data describe that the absence of the IP hampers the ability to induce effector T cells and affect CD8^+^ T cell differentiation into memory/effector T cells, since an increased proportion of T cells were differentiated into central memory cells (**Figure 7H**).

#### IP Deficiency Affects Apoptosis and Signaling *via* STAT3 in TKO Mice in Chronic *T. gondii* Infection

**Figure 8:**
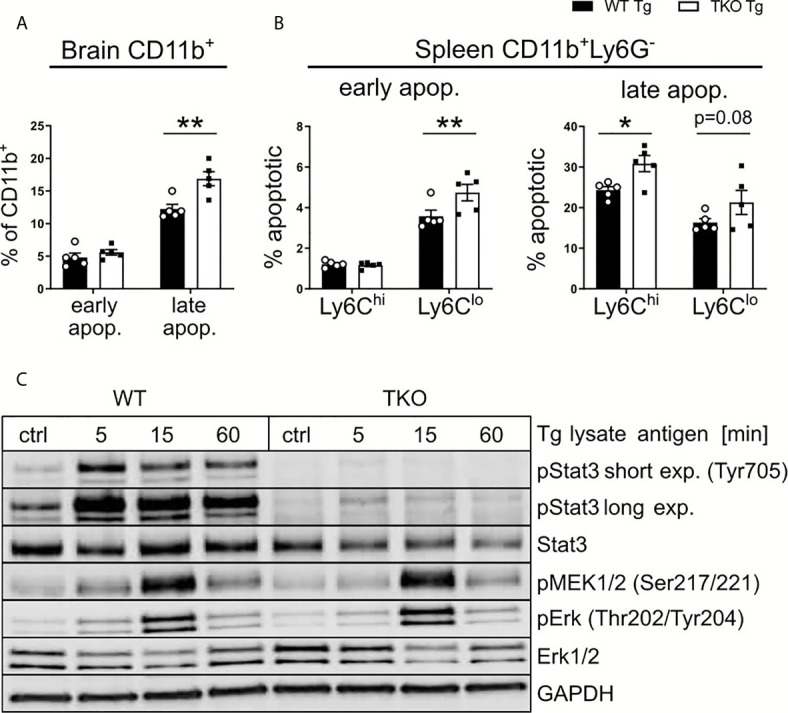
Altered STAT3 signaling in TKO APCs. Immune cells were isolated from the brain and spleen tissue of *T. gondii*-infected WT (WT Tg, n=5) and TKO (TKO Tg, n=5) mice on day 28 *p.i.* and analyzed by flow cytometry. **(A, B)** Isolated cells were stained with Annexin V and 7AAD to determine early apoptotic (7AAD^-^AnnexinV^+^) and late apoptotic (7AAD^+^AnnexinV^+^) cells. **(A)** Percentage of early and late apoptotic CD11b^+^ cells isolated from brain tissue. **(B)** Percentage of early and late apoptotic Ly6C^hi^ and Ly6C^lo^ mononuclear cells isolated from spleen. **(C)** Bone marrow derived macrophages from WT and TKO mice were treated with 30μg/ml *toxoplasma* lysate for the depicted time. Proteins were isolated and quantified *via* Bradford assay and immunoblotted using pMEK (Ser217/221), Erk, pErk (Thr202/Tyr204), Stat3, pStat3 (Tyr705) and GAPDH antibodies. For apoptosis assay, n=5. Data shown in **(A, B)** represent three independent experiments; symbols represent individual animals, columns represent mean values and error bars represent ± SEM. Data shown in **(C)** represents a representative of three independent experiments. 2way ANOVA followed by Fisher’s LSD test was used for statistical analysis. *P < 0.05, **P < 0.01.

Since *T. gondii* is known to infect APCs, DCs in particular, as well as the IP primarily seems to affect CD8^+^ T cell numbers by altered MHC I/peptide presentation, this suggests an important role for APCs in the brain in the chronic stage of infection. To further investigate this hypothesis, we determined the frequencies of apoptotic APCs in brain (**Figure 8A**) and spleen (**Figure 8B**) tissue of WT and TKO mice in the chronic stage of infection. Using Annexin V and 7AAD, we assessed early and late apoptotic APCs in infected animals in the chronic stage of infection. First analyzing CD11b^+^ cells (to include microglia) in brains from infected animals on day 28 *p.i.*, we detected comparable early apoptotic, but significantly increased frequencies of late apoptotic cells in TKO mice compared to WT mice (**Figure 8A**). Splenocytes were isolated from infected animals on day 28 *p.i.* and all CD11b^+^ splenocytes were further divided into Ly6C^hi^ and Ly6C^lo^ cells. We observed significantly increased frequencies of early apoptotic Ly6C^lo^ cells, whereas significantly increased frequencies of late apoptotic Ly6C^hi^ and Ly6C^lo^ cells were found (**Figure 8B**). Thus, with the absence of the IP, APCs in brain and spleen tissue of chronically infected animals have increased rates of apoptosis (**Figures 8A, B**). It is conceivable that this is a potential mechanism, explaining the observed reduced numbers of CD8^+^ T_em_ cells in brains of TKO mice (**Figures 7F’**).

During inflammation, the IP is a crucial component needed for cell signaling and protein degradation. Studies have hypothesized that the IP plays a role in regulating pro-inflammatory cytokines (42, 43). Thus, we aimed to determine if deficiency of the IP affects any major cytokine signaling pathways found in APCs such as MAPK/NF-κB or STAT pathways. These signaling pathways are known to be essentially involved in *T. gondii* containment (35, 44, 45) and further can be manipulated by the parasite itself thereby using them to evade the host immune system (46, 47). Bone marrow derived macrophages (BMDMs) were stimulated with TLA *ex vivo* and protein expression was analyzed *via* immunoblot (**Figures 8C**). We analyzed different key proteins from different stages of the MAPK/NF-κB pathway. No differences in the phosphorylation of MEK and ERK could be detected between WT and TKO mice following stimulation. We further analyzed STAT3 and its phosphorylated variant (pSTAT3) as a key component of the STAT pathway. BMDMs of TKO mice compared to WT mice showed a marked reduction in STAT3 phosphorylation. It is described that STAT3, and subsequent pSTAT3, are crucial components for cell survival and IL-6/10/12 signaling (48–50). This finding fits to our observation of increased apoptosis in brains of TKO mice in the chronic stage of infection. These data highlight that the absence of the IP impairs STAT3 signaling *via* dysregulated phosphorylation (**Figure 8C** and **Supplementary Figures 5A, B**), correlating with the observed reduced myeloid cell survival (**Figures 8A, B**) and altered T cell differentiation (**Figures 7F, F’**) in infected TKO mice in the chronic stage of infection.

## Discussion

The results presented in the current study demonstrate that the IP is a crucial component of the immune system for the transition between innate and adaptive immune responses against *T. gondii.* The absence of the IP subunits LMP2, MECL-1 and LMP7 indirectly showed a reduced ability of APCs to present peptides to T cells by displaying decreased MHC I cell surface level, thereby reducing the pool of the available CD8^+^ T cells, all crucial steps for *T. gondii* containment and clearance. Furthermore, these APCs were more prone to apoptosis and lacked STAT3 phosphorylation. Ultimately, this impaired immune response lead to an inability of TKO mice to control parasite proliferation, causing reactivation of toxoplasmosis resulting in an increased susceptibility of TKO mice in a *T. gondii* infection model.

TKO mice showed an increased weight loss during the chronic course of *T. gondii* infection that is often associated with an enhanced immune response. And in fact, brain tissue of chronically infected TKO mice showed increased TNF and IFNγ as well as increased production of these cytokines released by CD4^+^ T cells in the chronic phase of infection. Nevertheless, *T. gondii* infected TKO mice showed an inability to control the parasite burden, particularly, in the acute phase but also in the chronic phase of infection. This inability for early parasite containment is presumably caused by a delayed antigen presentation by APCs. Dysregulated antigen presentation by APCs can delay parasite specific T cell activation and proliferation thereby delaying expression of IFNγ induced anti-parasitic effector molecules. This mechanism aligns with other infection models using TKO animals. Infection with *Brucella abortus* in TKO mice led to an increased bacterial burden. This was associated with an impaired MHC I presentation of CD11c^+^ cells and a reduced percentage of both CD4^+^ and CD8^+^ IFNγ producing T cells as well as fewer Granzyme B producing CD8^+^ T cells (37). Similarly, infection with the protozaon *Trypanosoma cruzi* in TKO mice resulted in reduced MHC I expression and altered CD8^+^ effector T cell function, in both quantity and quality as there were fewer overall CD8^+^ effector cells and fewer IFNγ producers (36). However, depending on the pathogen type, its organ specificity and impaired IP subunit expression as well as the duration of the challenge, the IP’s contribution varies.

In a *Leishmania major* infection model, the absence of the subunit LMP7 had no effect on the ability of DCs to stimulate CD8^+^ T cells in both WT and LMP7^-/-^ mice, as well as the authors showed similar IFNγ production and T cell proliferation (51). The role of LMP7 was further highlighted in a malaria infection model, since the absence of LMP7 resulted in lower parasite growth, reduced parasite burden but an enhanced immune response with increased phagocytosis activity (52). LMP7^-/-^ mice displayed reduced MHC I expression on APCs (53) and infected LMP2^-/-^ mice showed a strong reduction (~70%) of CD8^+^ lymphocytes compared to WT mice (54). In addition, MECL-1^-/-^ mice similar to LMP2^-/-^ mice, showed a reduction of CD8^+^ T cells in the spleen compared to WT mice (55), at which MECL-1 contributes to T cell homeostatic expansion (56). Notably, using the LCMV infection model, Nussbaum et al., observed that although LMP2^-/-^ or LMP7^-/-^ mice had fewer CD8^+^ T cells, these animals were able to mount strong CD8^+^ anti-viral immune responses demonstrated by similar kinetics of viral clearance compared to WT mice (57). In addition, analyzing the role of mouse adenovirus type 1 infection in pathogenesis of TKO mice the authors detected age-dependent differing effects (58). All these studies demonstrate that the role of the IP during infection is multifaceted and most likely pathogen specific.

DCs and Ly6C^hi^ monocytes in spleens of acutely infected TKO mice possessed a slightly increased production of TNF but not IL-12, indicating that parasite detection was still intact. However, Ly6C^hi^ monocytes and DCs from TKO mice showed reduced cell numbers with impaired MHC I expression in spleen and brain tissue during both the acute and the chronic stage of infection. This reduced recruitment of APCs to the sites of infection not only delays IFNγ induced T cell priming, but also leads to a delayed initiation of the adaptive immune response as fewer APCs are able to present parasite specific antigens. Thus, in the acute phase of infection an attenuated inflammation can be detected which is similar to the phenotype observed in models of autoimmune-related myocarditis and experimental autoimmune encephalomyelitis due to immunoproteasome inhibition (59, 60). In contrast, an opposite scenario could be observed during the chronic stage of infection where Ly6C^hi^ monocytes and DCs could be found in the brain of TKO mice which released higher levels of TNF and iNOS. In addition, proinflammatory cytokines were increased in whole brain homogenates of chronically infected TKO mice. These results indicate a dysregulated immune response to *T. gondii*. In the absence of the immunoproteasome, an efficient immune response cannot be initiated during the acute phase of infection. Further, the resulting excessive inflammatory response in the chronic phase is insufficient to efficiently control the infection. This is in concordance with previously published data showing that IP-formation is crucial for protection from virus-induced inflammatory tissue damage as observed in coxsackievirus B3 myocarditis (27). Notably, enhanced NF-κB activity and TNF production can be mediated even in the absence of immunoproteasomes as observed in our study e.g. by increasing the degradation of the NF-κB inhibitor IκBα through 20S proteasome complexes associated with the proteasome activator PA28 that is constitutively expressed in various tissues (61, 62).

An impaired MHC I-antigen peptide activation of CD8^+^ T cells is in line with previous results illustrating the pivotal role of the IP subunit LMP7 during *T. gondii* infection in regard to induction of DC driven activation of cytotoxic CD8^+^ T cells (24). Furthermore, mice deficient for the single IP subunits LMP2 or LMP7 showed increased susceptibility to *T. gondii* infection and displayed less IFNγ-secreting CD8^+^ T cells following infection although they had similar numbers of activated CD8^+^ T cells compared to WT mice (24). It should be noted that in our study a lower dose of *T. gondii* as well as a different infection route was used, thus reducing inflammation that resulted in reduced susceptibility of TKO mice compared to single subunit knock out mice in *T. gondii* infection (24).

As described above, *T. gondii* infected TKO mice showed a clearly reduced capability of APCs for antigen presentation, further suggesting a delayed induction of a Th1 adaptive immune response to *T. gondii* in TKO mice. And in indeed, we observed reduced numbers of CD8^+^ T cells as well as IFNγ producing CD4^+^ T cells in spleens of infected TKO mice in the acute phase of *T. gondii* infection, whereby parasite proliferation is not restricted properly. In addition, we detected increased numbers of NK1.1^+^ cells in brains of infected TKO mice which could possibly compensate for the absence of activated CD8^+^ T cells.

Similar to the NK1.1^+^ cells in brains of TKO mice in the acute phase of infection, it seems that CD4^+^ T cells in the brain of TKO mice in the chronic phase of infection could compensate for the reduced CD8^+^ T cell response. We found significantly more IFNγ and TNF producing CD4^+^ T cells in brains of infected TKO mice in the chronic stage of infection. This correlates with an increase in iNOS production in mononuclear cells. Given the fact that iNOS is a crucial anti-parasitic effector molecule during chronic infection (63), it could compensate in part for the lack of CD8-mediated intracellular parasite clearance in the brain. In contrast, TKO mice exhibited reduced CD8^+^ Tem cells in the chronic stage of infection suggesting that the absence of the IP hampers the ability to induce effector T cells timely after infectious challenge.

Regulatory T cells (Tregs), as a subpopulation of T cells, are important to suppress T cell function to regulate self-tolerance thereby preventing autoimmunity (64). We hypothesized that fewer Tregs would affect the contraction phase of the T cell response. Usually, the contraction phase begins once the pathogen has been cleared. This in turn leads to the upregulation of exhaustion markers resulting in apoptosis (65–67). Although parasites are still present, it is possible that the reduced MHC I/TCR signaling leads to reduced CD8^+^ T cell interaction with their associated antigen, thus behaving as if there is no pathogen present, ultimately starting exhaustion earlier than anticipated. Infected TKO mice, however, showed comparable expression of T cell exhaustion and apoptosis markers in CD8^+^ and CD4^+^ T cells (**Supplementary Figure 4**). Further, we found increased numbers of apoptotic monocytes in spleens of TKO mice in the chronic phase of infection. This could be explained by the inability of TKO derived myeloid cells to induce STAT3-signaling by its phosphorylation, a mechanism which has also been described in Th17 cells after IP inhibition (68). Consistent with this finding, STAT3-deficiency in B lymphocytes has been shown to induce apoptosis in a model of experimental autoimmune uveitis (69). However, it still has to be investigated whether the observed apoptosis is caused by direct parasite invasion or by the absence of the IP itself.

In summary, our results established the importance of the IP in infection-induced neuroinflammation with *T. gondii*. Without the IP, animals were impeded in developing an efficient *T. gondii* specific Th1 immune response. With reduced MHC I expression, CD8^+^ T cell numbers and IFNγ in the acute phase, TKO mice were not able to control parasite proliferation, especially by their inability to promote the transition of the acute phase to an efficient long lasting immune response during the chronic stage of *T. gondii* infection.

We described an enhanced compensatory CD4^+^ T cell effector function in TKO mice with increased IFNγ release during the course of infection. In addition, we detected increased production of iNOS in microglia and myeloid subsets and overall enhanced TNF level in brain tissue of chronically infected TKO animals as well as reduced numbers of regulatory T cells, reduced STAT3 phosphorylation but increased induction of apoptosis in myeloid cells. This study demonstrates that IP deficiency leads to impaired parasite control and thus increased susceptibility of these animals to *T. gondii*, highlighting the importance of the IP in terms of induction and maintenance of *T. gondii*-induced neuroinflammation.

## Data Availability Statement

The original contributions presented in the study are included in the article/**Supplementary Material**. Further inquiries can be directed to the corresponding author.

## Ethics Statement

The study was performed in accordance with the German National Guidelines for the Use of Experimental Animals and the protocol was approved by the Landesverwaltungsamt Sachsen-Anhalt. Food and water were available *ad libitum*. All efforts were done to minimize the suffering of mice used in this investigation. The animal study was reviewed and approved by German and European legislation.

## Author Contributions

TF and IRD designed and organized the experiments. TF, NI, HD, CC, and ET conducted the experiments. TF and NI analyzed data. TF, AT, JS, DD, TS, US, and IRD interpreted data. TF, TS, US and IRD wrote the paper. US and IRD supervised the study. All authors contributed to the article and approved the submitted version.

## Funding

This work was supported by the European Structural and Investment Funds (ESF, 2014-2020; project number ZS/2016/08/80645 to IRD and the DFG (Deutsche Forschungsgemeinschaft) within the CRC854 to DD, US and IRD. We acknowledge support for the Article Processing Charge from the DFG (German Research Foundation, 393148499) and the Open Access Publication Fund of the University of Greifswald.

## Conflict of Interest

The authors declare that the research was conducted in the absence of any commercial or financial relationships that could be construed as a potential conflict of interest.

## References

[1] Wilking H Thamm M Stark K Aebischer T Seeber F. Prevalence, incidence estimations, and risk factors of Toxoplasma gondii infection in Germany: a representative, cross-sectional, serological study. Sci Rep (2016) 6:22551. 10.1038/srep22551 26936108 PMC4776094

[2] Liu L Liu L-N Wang P Lv T-T Fan Y-G Pan H-F. Elevated seroprevalence of Toxoplasma gondii in AIDS/HIV patients: A meta-analysis. Acta Trop (2017) 176:162–7. 10.1016/j.actatropica.2017.08.001 28784422

[3] Dunay IR Fuchs A Sibley LD. Inflammatory monocytes but not neutrophils are necessary to control infection with Toxoplasma gondii in mice. Infect Immun (2010) 78:1564–70. 10.1128/IAI.00472-09 PMC2849397 20145099

[4] Mashayekhi M Sandau MM Dunay IR Frickel EM Khan A Goldszmid RS. CD8α(+) dendritic cells are the critical source of interleukin-12 that controls acute infection by Toxoplasma gondii tachyzoites. Immunity (2011) 35:249–59. 10.1016/j.immuni.2011.08.008 PMC3171793 21867928

[5] Matta SK Rinkenberger N Dunay IR Sibley LD. Toxoplasma gondii infection and its implications within the central nervous system. Nat Rev Microbiol (2021). 10.1038/s41579-021-00518-7 33627834

[6] Yarovinsky F. Innate immunity to Toxoplasma gondii infection. Nat Rev Immunol (2014) 14:109–21. 10.1038/nri3598 24457485

[7] Suzuki Y Wang X Jortner BS Payne L Ni Y Michie SA. Removal of Toxoplasma gondii cysts from the brain by perforin-mediated activity of CD8+ T cells. Am J Pathol (2010) 176:1607–13. 10.2353/ajpath.2010.090825 PMC2843452 20167872

[8] Pittman KJ Knoll LJ. Long-Term Relationships: the Complicated Interplay between the Host and the Developmental Stages of Toxoplasma gondii during Acute and Chronic Infections. Microbiol Mol Biol Rev (2015) 79:387–401. 10.1128/MMBR.00027-15 26335719 PMC4557073

[9] Gazzinelli R Xu Y Hieny S Cheever A Sher A. Simultaneous depletion of CD4+ and CD8+ T lymphocytes is required to reactivate chronic infection with Toxoplasma gondii. J Immunol (1992) 149:175–80. 1351500

[10] Khan IA Kasper LH. IL-15 augments CD8+ T cell-mediated immunity against Toxoplasma gondii infection in mice. J Immunol (1996) 157:2103–8. 8757333

[11] Khan IA Hwang S Moretto M. Toxoplasma gondii: CD8 T Cells Cry for CD4 Help. Front Cell Infect Microbiol (2019) 9:136. 10.3389/fcimb.2019.00136 31119107 PMC6504686

[12] Gazzinelli RT Hakim FT Hieny S Shearer GM Sher A. Synergistic role of CD4+ and CD8+ T lymphocytes in IFN-gamma production and protective immunity induced by an attenuated Toxoplasma gondii vaccine. J Immunol (1991) 146:286–92. 1670604

[13] Strehl B Seifert U Krüger E Heink S Kuckelkorn U Kloetzel P-M. Interferon-gamma, the functional plasticity of the ubiquitin-proteasome system, and MHC class I antigen processing. Immunol Rev (2005) 207:19–30. 10.1111/j.0105-2896.2005.00308.x 16181324

[14] Ebstein F Kloetzel P-M Krüger E Seifert U. Emerging roles of immunoproteasomes beyond MHC class I antigen processing. Cell Mol Life Sci (2012) 69:2543–58. 10.1007/s00018-012-0938-0 PMC11114860 22382925

[15] Kincaid EZ Che JW York I Escobar H Reyes-Vargas E Delgado JC. Mice completely lacking immunoproteasomes display major alterations in antigen presentation. Nat Immunol (2011) 13:129–35. 10.1038/ni.2203 PMC3262888 22197977

[16] Seifert U Bialy LP Ebstein F Bech-Otschir D Voigt A Schröter F. Immunoproteasomes preserve protein homeostasis upon interferon-induced oxidative stress. Cell (2010) 142:613–24. 10.1016/j.cell.2010.07.036 20723761

[17] Toes RE Nussbaum AK Degermann S Schirle M Emmerich NP Kraft M. Discrete cleavage motifs of constitutive and immunoproteasomes revealed by quantitative analysis of cleavage products. J Exp Med (2001) 194:1–12. 10.1084/jem.194.1.1 11435468 PMC2193442

[18] Chen X Zhang X Wang Y Lei H Su H Zeng J. Inhibition of immunoproteasome reduces infarction volume and attenuates inflammatory reaction in a rat model of ischemic stroke. Cell Death Dis (2015) 6:e1626. 10.1038/cddis.2014.586 25633295 PMC4669779

[19] Orre M Kamphuis W Dooves S Kooijman L Chan ET Kirk CJ. Reactive glia show increased immunoproteasome activity in Alzheimer’s disease. Brain (2013) 136:1415–31. 10.1093/brain/awt083 23604491

[20] Moritz KE McCormack NM Abera MB Viollet C Yauger YJ Sukumar G. The role of the immunoproteasome in interferon-γ-mediated microglial activation. Sci Rep (2017) 7:9365. 10.1038/s41598-017-09715-y 28839214 PMC5571106

[21] Fischer R Maier O. Interrelation of oxidative stress and inflammation in neurodegenerative disease: role of TNF. Oxid Med Cell Longev (2015) 2015:610813. 10.1155/2015/610813 25834699 PMC4365363

[22] Brown GC Neher JJ. Inflammatory neurodegeneration and mechanisms of microglial killing of neurons. Mol Neurobiol (2010) 41:242–7. 10.1007/s12035-010-8105-9 20195798

[23] Mundt S Engelhardt B Kirk CJ Groettrup M Basler M. Inhibition and deficiency of the immunoproteasome subunit LMP7 attenuates LCMV-induced meningitis. Eur J Immunol (2016) 46:104–13. 10.1002/eji.201545578 26464284

[24] Tu L Moriya C Imai T Ishida H Tetsutani K Duan X. Critical role for the immunoproteasome subunit LMP7 in the resistance of mice to Toxoplasma gondii infection. Eur J Immunol (2009) 39:3385–94. 10.1002/eji.200839117 19830724

[25] Parlog A Harsan L-A Zagrebelsky M Weller M von Elverfeldt D Mawrin C. Chronic murine toxoplasmosis is defined by subtle changes in neuronal connectivity. Dis Model Mech (2014) 7:459–69. 10.1242/dmm.014183 PMC3974456 24524910

[26] Möhle L Israel N Paarmann K Krohn M Pietkiewicz S Müller A. Chronic Toxoplasma gondii infection enhances β-amyloid phagocytosis and clearance by recruited monocytes. Acta Neuropathol Commun (2016) 4:25. 10.1186/s40478-016-0293-8 26984535 PMC4793516

[27] Opitz E Koch A Klingel K Schmidt F Prokop S Rahnefeld A. Impairment of immunoproteasome function by β5i/LMP7 subunit deficiency results in severe enterovirus myocarditis. PloS Pathog (2011) 7:e1002233. 10.1371/journal.ppat.1002233 21909276 PMC3164653

[28] Möhle L Parlog A Pahnke J Dunay IR. Spinal cord pathology in chronic experimental Toxoplasma gondii infection. Eur J Microbiol Immunol (Bp) (2014) 4:65–75. 10.1556/EuJMI.4.2014.1.6 24678407 PMC3955833

[29] Bereswill S Kühl AA Alutis M Fischer A Möhle L Struck D. The impact of Toll-like-receptor-9 on intestinal microbiota composition and extra-intestinal sequelae in experimental Toxoplasma gondii induced ileitis. Gut Pathog (2014) 6:19. 10.1186/1757-4749-6-19 24932221 PMC4057803

[30] Lehmann JS Zhao A Sun B Jiang W Ji S. Multiplex Cytokine Profiling of Stimulated Mouse Splenocytes Using a Cytometric Bead-based Immunoassay Platform. J Vis Exp (2017) 129:e56440. 10.3791/56440 PMC5755345 29155764

[31] Lambert H Barragan A. Modelling parasite dissemination: host cell subversion and immune evasion by Toxoplasma gondii. Cell Microbiol (2010) 12:292–300. 10.1111/j.1462-5822.2009.01417.x 19995386

[32] Wilson DC Grotenbreg GM Liu K Zhao Y Frickel E-M Gubbels M-J. Differential regulation of effector- and central-memory responses to Toxoplasma gondii Infection by IL-12 revealed by tracking of Tgd057-specific CD8+ T cells. PloS Pathog (2010) 6:e1000815. 10.1371/journal.ppat.1000815 20333242 PMC2841619

[33] Takács AC Swierzy IJ Lüder CG. Interferon-γ Restricts Toxoplasma gondii Development in Murine Skeletal Muscle Cells via Nitric Oxide Production and Immunity-Related GTPases. PloS One (2012) 7:e45440. 10.1371/journal.pone.0045440 23024821 PMC3443239

[34] Zhao YO Khaminets A Hunn JP Howard JC. Disruption of the Toxoplasma gondii parasitophorous vacuole by IFNgamma-inducible immunity-related GTPases (IRG proteins) triggers necrotic cell death. PloS Pathog (2009) 5:e1000288. 10.1371/journal.ppat.1000288 19197351 PMC2629126

[35] Hunter CA Sibley LD. Modulation of innate immunity by Toxoplasma gondii virulence effectors. Nat Rev Microbiol (2012) 10:766–78. 10.1038/nrmicro2858 PMC3689224 23070557

[36] Ersching J Vasconcelos JR Ferreira CP Caetano BC Machado AV Bruna-Romero O. The Combined Deficiency of Immunoproteasome Subunits Affects Both the Magnitude and Quality of Pathogen- and Genetic Vaccination-Induced CD8+ T Cell Responses to the Human Protozoan Parasite Trypanosoma cruzi. PloS Pathog (2016) 12:e1005593. 10.1371/journal.ppat.1005593 27128676 PMC4851296

[37] Guimarães G Gomes MT Campos PC Marinho FV de Assis NR Silveira TN. Immunoproteasome Subunits Are Required for CD8+ T Cell Function and Host Resistance to Brucella abortus Infection in Mice. Infect Immun (2018) 86(3):e00617–17. 10.1128/IAI.00615-17 PMC5820958 29263103

[38] Harker KS Ueno N Lodoen MB. Toxoplasma gondii dissemination: a parasite’s journey through the infected host. Parasite Immunol (2015) 37:141–9. 10.1111/pim.12163 25408224

[39] Biswas A Bruder D Wolf SA Jeron A Mack M Heimesaat MM. Ly6C(high) monocytes control cerebral toxoplasmosis. J Immunol (2015) 194:3223–35. 10.4049/jimmunol.1402037 25710908

[40] Kasper L Courret N Darche S Luangsay S Mennechet F Minns L. Toxoplasma gondii and mucosal immunity. Int J Parasitol (2004) 34:401–9. 10.1016/j.ijpara.2003.11.023 15003499

[41] Kincaid EZ Murata S Tanaka K Rock KL. Specialized proteasome subunits have an essential role in the thymic selection of CD8(+) T cells. Nat Immunol (2016) 17:938–45. 10.1038/ni.3480 PMC4955723 27294792

[42] Ebstein F Voigt A Lange N Warnatsch A Schröter F Prozorovski T. Immunoproteasomes are important for proteostasis in immune responses. Cell (2013) 152:935–7. 10.1016/j.cell.2013.02.018 23452842

[43] Visekruna A Joeris T Seidel D Kroesen A Loddenkemper C Zeitz M. Proteasome-mediated degradation of IkappaBalpha and processing of p105 in Crohn disease and ulcerative colitis. J Clin Invest (2006) 116:3195–203. 10.1172/JCI28804 PMC1654202 17124531

[44] Gavrilescu LC Butcher BA Del Rio L Taylor GA Denkers EY. STAT1 is essential for antimicrobial effector function but dispensable for gamma interferon production during Toxoplasma gondii infection. Infect Immun (2004) 72:1257–64. 10.1128/IAI.72.3.1257-1264.2004 PMC356043 14977926

[45] Harris TH Wilson EH Tait ED Buckley M Shapira S Caamano J. NF-κB1 contributes to T cell-mediated control of Toxoplasma gondii in the CNS. J Neuroimmunol (2010) 222:19–28. 10.1016/j.jneuroim.2009.12.009 20156658 PMC2860689

[46] Laliberté J Carruthers VB. Host cell manipulation by the human pathogen Toxoplasma gondii. Cell Mol Life Sci (2008) 65:1900–15. 10.1007/s00018-008-7556-x PMC2662853 18327664

[47] Denkers EY Bzik DJ Fox BA Butcher BA. An inside job: hacking into Janus kinase/signal transducer and activator of transcription signaling cascades by the intracellular protozoan Toxoplasma gondii. Infect Immun (2012) 80:476–82. 10.1128/IAI.05974-11 PMC3264314 22104110

[48] Hirano T Ishihara K Hibi M. Roles of STAT3 in mediating the cell growth, differentiation and survival signals relayed through the IL-6 family of cytokine receptors. Oncogene (2000) 19:2548–56. 10.1038/sj.onc.1203551 10851053

[49] Hutchins AP Diez D Miranda-Saavedra D. The IL-10/STAT3-mediated anti-inflammatory response: recent developments and future challenges. Brief Funct Genomics (2013) 12:489–98. 10.1093/bfgp/elt028 PMC3838198 23943603

[50] Butcher BA Kim L Panopoulos AD Watowich SS Murray PJ Denkers EY. IL-10-independent STAT3 activation by Toxoplasma gondii mediates suppression of IL-12 and TNF-alpha in host macrophages. J Immunol (2005) 174:3148–52. 10.4049/jimmunol.174.6.3148 15749841

[51] Brosch S Tenzer S Akkad N Lorenz B Schild H von Stebut E. Priming of Leishmania-reactive CD8+ T cells in vivo does not require LMP7-containing immunoproteasomes. J Invest Dermatol (2012) 132:1302–5. 10.1038/jid.2011.454 22277939

[52] Duan X Imai T Chou B Tu L Himeno K Suzue K. Resistance to malaria by enhanced phagocytosis of erythrocytes in LMP7-deficient mice. PloS One (2013) 8:e59633. 10.1371/journal.pone.0059633 23527234 PMC3602297

[53] Fehling HJ Swat W Laplace C Kühn R Rajewsky K Müller U. MHC class I expression in mice lacking the proteasome subunit LMP-7. Science (1994) 265:1234–7. 10.1126/science.8066463 8066463

[54] van Kaert L Ashton-Rickardt PG Eichelberger M Gaczynska M Nagashima K Rock KL. Altered peptidase and viral-specific T cell response in LMP2 mutant mice. Immunity (1994) 1:533–41. 10.1016/1074-7613(94)90043-4 7600282

[55] Basler M Moebius J Elenich L Groettrup M Monaco JJ. An altered T cell repertoire in MECL-1-deficient mice. J Immunol (2006) 176:6665–72. 10.4049/jimmunol.176.11.6665 16709825

[56] Zaiss DM de Graaf N Sijts AJ. The proteasome immunosubunit multicatalytic endopeptidase complex-like 1 is a T-cell-intrinsic factor influencing homeostatic expansion. Infect Immun (2008) 76:1207–13. 10.1128/IAI.01134-07 PMC2258853 18160473

[57] Nussbaum AK Rodriguez-Carreno MP Benning N Botten J Whitton JL. Immunoproteasome-deficient mice mount largely normal CD8+ T cell responses to lymphocytic choriomeningitis virus infection and DNA vaccination. J Immunol (2005) 175:1153–60. 10.4049/jimmunol.175.2.1153 16002717

[58] Chandrasekaran A Adkins LJ Seltzer HM Pant K Tryban ST Molloy CT. Age-Dependent Effects of Immunoproteasome Deficiency on Mouse Adenovirus Type 1 Pathogenesis. J Virol (2019) 93(15):e00569–19. 10.1128/JVI.00569-19 PMC6639286 31092582

[59] Bockstahler M Fischer A Goetzke CC Neumaier HL Sauter M Kespohl M. Heart-Specific Immune Responses in an Animal Model of Autoimmune-Related Myocarditis Mitigated by an Immunoproteasome Inhibitor and Genetic Ablation. Circulation (2020) 141:1885–902. 10.1161/CIRCULATIONAHA.119.043171 32160764

[60] Basler M Mundt S Muchamuel T Moll C Jiang J Groettrup M. Inhibition of the immunoproteasome ameliorates experimental autoimmune encephalomyelitis. EMBO Mol Med (2014) 6:226–38. 10.1002/emmm.201303543 PMC3927957 24399752

[61] Mitchell S Mercado EL Adelaja A Ho JQ Cheng QJ Ghosh G. An NFκB Activity Calculator to Delineate Signaling Crosstalk: Type I and II Interferons Enhance NFκB via Distinct Mechanisms. Front Immunol (2019) 10:1425. 10.3389/fimmu.2019.01425 31293585 PMC6604663

[62] Keller M Ebstein F Bürger E Textoris-Taube K Gorny X Urban S. The proteasome immunosubunits, PA28 and ER-aminopeptidase 1 protect melanoma cells from efficient MART-126-35 -specific T-cell recognition. Eur J Immunol (2015) 45:3257–68. 10.1002/eji.201445243 26399368

[63] Scharton-Kersten TM Yap G Magram J Sher A. Inducible nitric oxide is essential for host control of persistent but not acute infection with the intracellular pathogen Toxoplasma gondii. J Exp Med (1997) 185:1261–73. 10.1084/jem.185.7.1261 PMC2196248 9104813

[64] Knochelmann HM Dwyer CJ Bailey SR Amaya SM Elston DM Mazza-McCrann JM. When worlds collide: Th17 and Treg cells in cancer and autoimmunity. Cell Mol Immunol (2018) 15:458–69. 10.1038/s41423-018-0004-4 PMC6068176 29563615

[65] Jin H-T Jeong YH Park HJ Ha S-J. Mechanism of T cell exhaustion in a chronic environment. BMB Rep (2011) 44:217–31. 10.5483/BMBRep.2011.44.4.217 21524346

[66] Bhadra R Gigley JP Khan IA. The CD8 T-cell road to immunotherapy of toxoplasmosis. Immunotherapy (2011) 3:789–801. 10.2217/imt.11.68 21668315 PMC4051159

[67] Duraiswamy J Kaluza KM Freeman GJ Coukos G. Dual blockade of PD-1 and CTLA-4 combined with tumor vaccine effectively restores T-cell rejection function in tumors. Cancer Res (2013) 73:3591–603. 10.1158/0008-5472.CAN-12-4100 PMC3686913 23633484

[68] Kalim KW Basler M Kirk CJ Groettrup M. Immunoproteasome subunit LMP7 deficiency and inhibition suppresses Th1 and Th17 but enhances regulatory T cell differentiation. J Immunol (2012) 189:4182–93. 10.4049/jimmunol.1201183 22984077

[69] Oladipupo FO Yu C-R Olumuyide E Jittaysothorn Y Choi JK Egwuagu CE. STAT3 deficiency in B cells exacerbates uveitis by promoting expansion of pathogenic lymphocytes and suppressing regulatory B cells (Bregs) and Tregs. Sci Rep (2020) 10:16188. 10.1038/s41598-020-73093-1 33004854 PMC7529787

