# Supplementary material for: The Immunoproteasome Subunits LMP2, LMP7 and MECL-1 Are Crucial Along the Induction of Cerebral Toxoplasmosis
Source: Front Immunol. 2021 Apr 21;12:619465. doi: 10.3389/fimmu.2021.619465 (PMC8099150; doi:10.3389/fimmu.2021.619465)
Supplement: Supplementary file 1 [file Image_1.pdf]

Supplementary Material

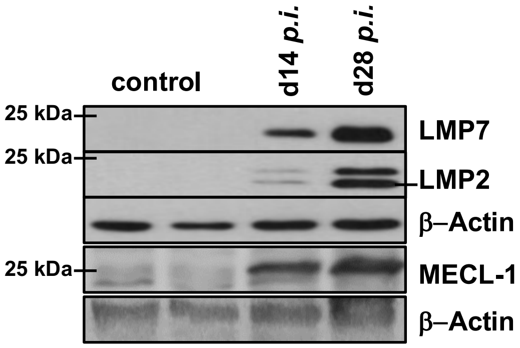

Supplementary Figure 1. Immunoproteasome subunit expression in acute and chronic stage of *T. gondii* infection

Proteins were isolated from the brain of naïve wild type (control) and *T. gondii*-infected (Tg) mice on d14 *p.i.* and d28 *p.i.* The proteins were quantified via Bradford assay and immunoblotted using β1i/LMP2, β2i/MECL-1 and β5i/LMP7 antibodies. β-Actin served as a loading control.

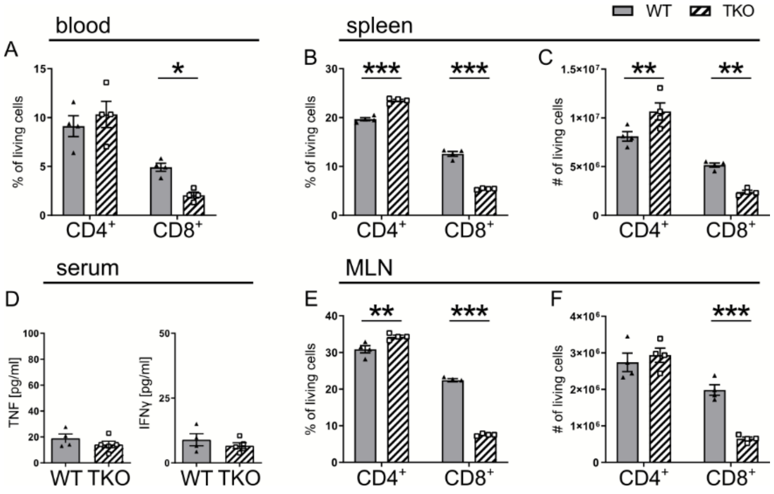

Supplementary Figure 2. Steady state immune populations in naïve WT and TKO animals

Immune cells were isolated from the blood, brain, spleen and mesenteric lymph node tissue from naïve WT (WT) and naïve TKO (TKO) mice and analyzed by flow cytometry. Following viability staining and the basic FSC/SSC gating, viable single cells were chosen for further characterization. Blood, MLN and splenocyte $CD11b^+$ cells were removed and $CD3^+CD4^+$ and $CD3^+CD8^+$ T cells were identified. The percentage of total living cells in (**A**) blood, (**B**) spleen, (**E**) MLN and the total number of living cells in (**C**) spleen and (**F**) MLN tissue were assessed. Serum was isolated from naïve WT and TKO mice. Using a cytokine bead array, the serum concentration of TNF and IFNγ were determined. Data in **A-C, E-F** represents three independent experiments. Data in **D** represents two independent experiments. Symbols represent individual animals; columns represent means and error bars represent ±SEM. In **D** a Mann-Whitney test for two groups and in **A-C, E-F** a 2way ANOVA followed by Fisher's LSD test was used for statistical analysis. *P<0.05, **P<0.01, ***P<0.001.

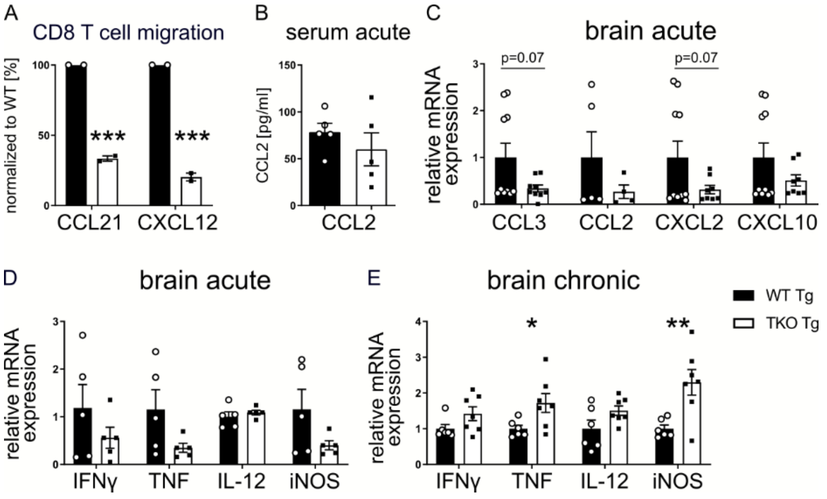

Supplementary Figure 3. Brain inflammatory marker gene expression

(**A**) $CD8^+$ T cells of naïve WT and TKO mice were magnetically sorted and transwell migration assay towards chemoattractants CXCL12 and CCL21 using 250 ng/mL was performed. Migrated cells are displayed as percentage of migrated WT control cells. Cells were quantified by flow cytometry. (**B**) Serum was isolated from *T. gondii*-infected WT (WT Tg, n=4) and TKO (TKO Tg, n=5) mice on day 10 p.i. Using a cytokine bead array, the serum concentration of CCL2 was determined. The brains were taken from acute (d10 p.i.) and chronic (d28 p.i.) *T. gondii*-infected WT (WT Tg) and TKO (TKO Tg) mice, homogenized and RNA was isolated for qRT-PCR analysis. Relative gene expression from the acute (d10 p.i.) and chronic (d28 p.i.) phase of infection for (**A**) chemokine and (**D,E**) Th1 inflammation markers. Relative mRNA levels were first normalized to housekeeping gene, HPRT, and then normalized to the mean expression of the WT Tg group. Data shown in **A,B** represents two independent experiments. Data shown in **C-E** represents three independent experiments, symbols represent individual animals, columns represent mean values and error bars represent ± SEM. In **B**, a Mann-Whitney test for two groups and in **A**, **C-E** a 2way ANOVA followed by Fisher's LSD test was used for statistical analysis. $*P<0.05$, $***P<0.001$.

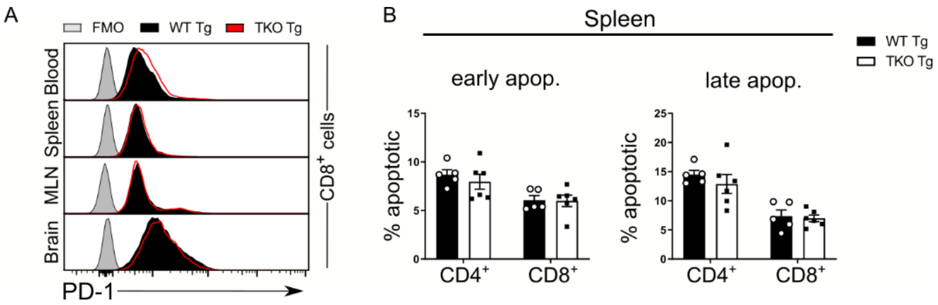

Supplementary Figure 4. CD8+ T cell exhaustion and apoptosis

Immune cells were isolated from the blood, spleen, MLN and brain tissue of *T. gondii*-infected WT (WT Tg, n=5) and TKO (TKO Tg, n=6) mice on day 28 *p.i.* and analyzed by flow cytometry. (**A**) To determine the exhaustion status of $CD8^+$ T cells, PD-1 surface expression MFI was analyzed. The histogram fluorescence minus one (FMO) control in gray, WT *T. gondii*-infected in black and TKO *T. gondii*-infected in red (**B**) $CD4^+$ and $CD8^+$ T cells were stained with Annexin V and 7AAD to determine early apoptotic ($7AAD^-AnnexinV^+$) and late apoptotic ($7AAD^+AnnexinV^+$) cells. Data shown represent three independent experiments; symbols represent individual animals, columns represent mean values and error bars represent ± SEM, 2way ANOVA followed by Fisher's LSD test was used for statistical analysis.

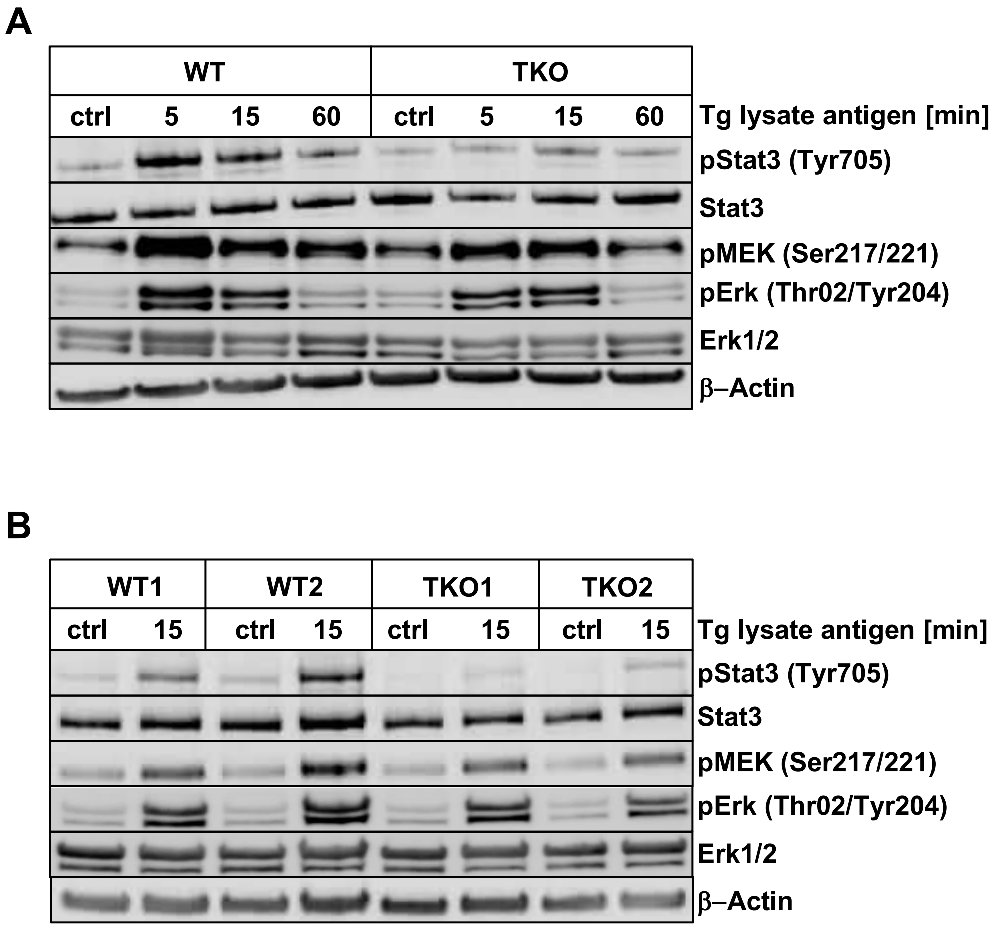

Supplementary Figure 5. Altered STAT3 signaling in immunoproteasome deficient APCs

Bone marrow derived macrophages from WT and TKO mice were treated with 30µg/ml (A) or 20 µg/ml (B) *toxoplasma* lysate for the depicted time. Proteins were isolated and quantified via Bradford assay and immunoblotted using pMEK (Ser217/221), Erk, pErk (Thr202/Tyr204), Stat3, pStat3 (Tyr705) and GAPDH antibodies. The blots are repetitions of Fig. 8C, two of three independent experiments.
